# Restriction and modification of deoxyarchaeosine (dG^+^)-containing phage 9 g DNA

**DOI:** 10.1038/s41598-017-08864-4

**Published:** 2017-08-21

**Authors:** Rebecca Tsai, Ivan R. Corrêa, Michael Y. Xu, Shuang-yong Xu

**Affiliations:** 1 New England Biolabs, Inc. 240 County Road, Ipswich, MA 01938 USA; 2 Present Address: Brandeis University, 415 South St., Waltham, MA 02453 USA

## Abstract

*E*. *coli* phage 9 g contains the modified base deoxyarchaeosine (dG^+^) in its genome. The phage encodes its own primase, DNA ligase, DNA polymerase, and enzymes necessary to synthesize and incorporate dG^+^. Here we report phage 9 g DNA sensitivity to >200 Type II restriction endonucleases (REases). Among the REases tested approximately 29% generated complete or partial digestions, while the remaining 71% displayed resistance to restriction. Phage 9 g restriction fragments can be degraded by DNA exonucleases or ligated by T3 and T4 DNA ligases. In addition, we examined a number of cytosine and adenine methyltransferases to generate double base modifications. M.AluI, M.CviPI, M.HhaI, and M.EcoGII were able to introduce ^5m^C or ^*N*6m^A into 9 g DNA as confirmed by partial resistance to restriction and by liquid chromatography-mass spectrometry. A number of wild-type *E*. *coli* bacteria restricted phage 9 g, indicating natural restriction barriers exist in some strains. A BlastP search of GenBank sequences revealed five glutamine amidotransferase-QueC homologs in *Enterobacteria* and *Pseudomonas* phage, and distant homologs in other phage and bacterial genomes, suggesting that dG^+^ is not a rare modification. We also mapped phage 9 g DNA packaging (*pac*) site containing two 21-bp direct repeats and a major terminase cleavage site in the phage genome.

## Introduction

In the restriction and anti-restriction arms race between bacteria and bacteriophage, phage have evolved elaborate DNA modifications on their genomes to evade host-encoded restriction endonucleases (REases) that attack phage DNA during infection^1^. Among the extensively studied phage DNA modifications are 5-methylcytosine (^5m^C) in phage XP12^2, 3^; 5-hydroxymethylcytosine (^5hm^C) in phage T4gt, glucosylated-5-hydroxymethylcytosine (^glc-5hm^C) in phage T4^4, 5^, *N*6-methyladenine (^*N*6m^A) in *Haemophilus influenzae* Rd prophage^6^; *N*6-(1-acetamido)-adenine in phage Mu^7, 8^; α-putrescinylthymine in φW-14^9, 10^; and 5-hydroxymethyluracil (^5hm^U) in *Bacillus* SP8^11, 12^. Strikingly, only ~15% of N6-(1-acetamido)-adenine in phage Mu renders its gDNA resistant to many Type I and II restriction systems of the host^8^. Another example of phage DNA modification is 2′-deoxyarchaeosine (abbreviated as dG^+^) in *E*. *coli* phage 9 g genome^13, 14^. Based on the conserved amino acid (aa) sequence prediction, the phage 9 g genome encodes its own primase, RNase H, DNA ligase, DNA polymerase, and 5–6 enzymes necessary to produce and incorporate the modified base and for DNA replication^13^. It has been estimated that the percentage of replacement of the standard guanine base with dG^+^ is in the range of 25–27% in the genome^14^. Among the seven REases tested previously on the phage 9 g DNA, five failed to cleave, implying that dG^+^ renders the 9 g DNA resistant to multiple Type II restriction systems^13^. The dG^+^ modification has also been detected or postulated in other *Enterobacteria* and *Pseudomonas* phages as well as in bacterial genomic islands based on gene clusters for dG^+^ synthesis and incorporation^14–16^.

Recently a ~20 kb gene cluster (genomic island, termed *dpd* cluster) encoding bacterial tRNA-guanine transglycosylase (i.e. TgtA5 encoded by *tgtA5* gene), 7-cyano-7-deazaguanine synthase (preQ_0_), and other DNA metabolic enzymes, including ATPase, DNA helicase, and a PLD-family endonuclease was found in *Samonella enterica* subsp. serovar Montevideo, *Kineococcus radiotolerans*, and other bacteria^14^. Several 7-deazaguanine derivatives were also detected from the *S*. *enterica* subsp. serovar Montevideo genomic DNA by LC-MS analysis, including 2′-deoxy-7-cyano-7-deazaguanine (dpreQ_0_), 2′-deoxy-7-aminomethyl-7-deazaguanine (dpreQ_1_), 2′-deoxy-7-amidoguanine (dADG), and 2′-deoxy-5-carboxydeazaguanine (dCDG). Moreover, homologs of TgtA5-like enzyme were found in more than 280 complete bacterial genome and uncultured bacteria. TgtA5 is similar but divergent from bacterial tRNA-guanine transglycosylase (bTGT), the enzyme that inserts preQ_1_ into tRNA at position 34 for queuosine-tRNA modification in bacteria. In addition, the *dpd* gene cluster encodes an active restriction system that restricted unmodified DNA^14^. The sequence specificity of the dG^+^ modification system if any is still unknown.

Since the modified base dG^+^ in phage DNA has only been discovered recently, it is not known whether the dG^+^-containing DNA can be further modified by other DNA modification enzymes, such as ^5m^C or ^N6m^A methyltransferases (MTases). It is also not clear whether the dG^+^-containing DNA can serve as a template in PCR by Phusion® and Q5® DNA polymerases. If phage 9 g is digested by Type II REases into small fragments, which DNA ligase can be used to ligate the sticky or blunt ends? Another interesting question is whether *E*. *coli*, *Enterobacteria*, and *Pseudomonas* hosts have evolved modification-dependent REases that specifically target the modified base dG^+^ in an analogous fashion to the host-encoded restriction enzyme GmrSD, which specifically targets the modified bases^5hm^C or ^glc-5hm^C on phage T4gt and T4 DNA^17–19^, the plasmid-encoded restriction system PvuRts1I, which prefers to attack ^5hm^C-containing DNA^20^, the host encoded SauUSI endonuclease that restricted ^5m^C-modified DNA^21^, and the ScoMcrA restricting phosphorothioated and ^m5^C-modified DNA^22^.

Here we report susceptibility of phage 9 g DNA to digestion by over 200 Type II REases. We examined the PCR efficiency of phage 9 g as a template and the ligation efficiency of three DNA ligases on 9 g restriction fragments. We also tested the activity of a number of DNA MTases on dG^+^-containing DNA. We studied phage 9 g restriction by a number of environmental *E*. *coli* strains. With bioinformatics tools we also looked at DNA/RNA metabolic enzymes, proteases, toxin and antitoxin associated with aTGT, bTGT, and TgtA5. Lastly, we mapped the *pac* site and the physical end of phage 9 g genome. This work aims at contributing to a better understanding of restriction and modification of dG^+^-containing DNA.

## Results

### dG^+^ composition in phage 9 g gDNA

**Figure 1:**
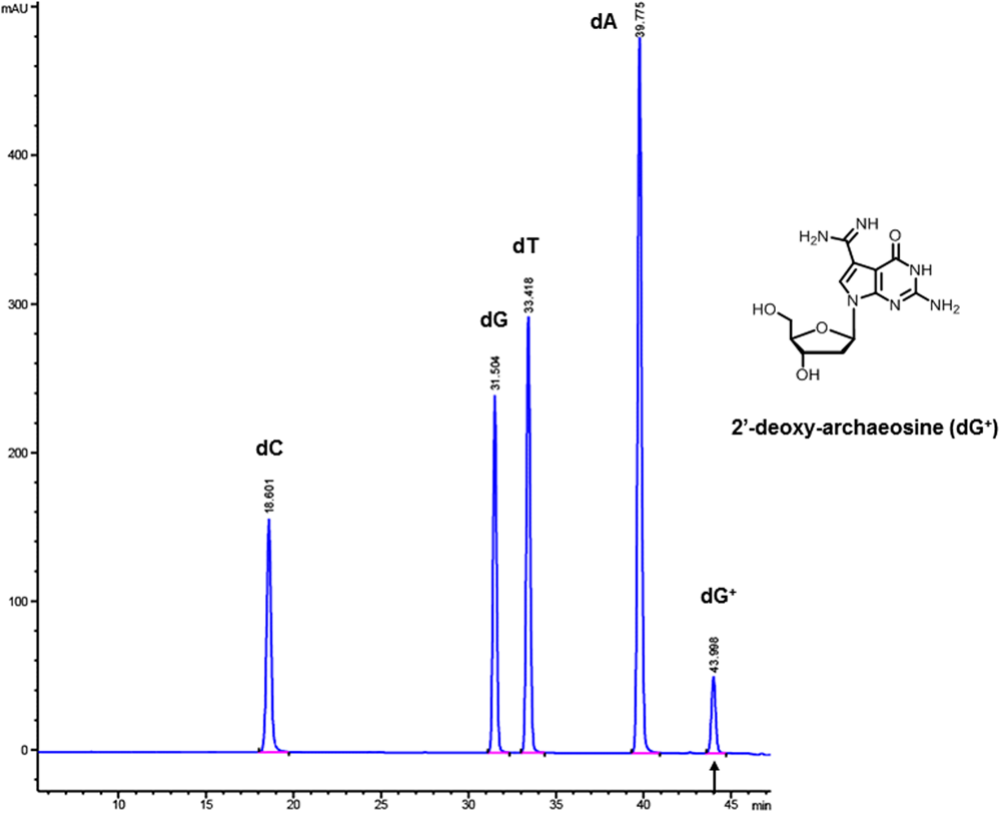
LC-MS analysis of phage 9 g base composition with modified base 2′-deoxyarchaeosine (dG^+^). The modified base dG^+^ is estimated at approximately 25–27% of total dG in the phage 9 g genome. The relative abundance of each nucleoside was determined by dividing the UV absorbance by the corresponding extinction coefficient at 260 nm. dG^+^ was quantified on the basis of the preQ_0_ extinction coefficient ε264 = 10,090 M^−1^ cm^−1^.

We confirmed the presence of the modified base dG^+^ in phage 9 g gDNA by non-specific nuclease degradation and dephosphorylation of DNA to mononucleosides, followed by LC-MS analysis. To ensure an accurate measurement of the dG^+^ composition, we performed gel purification of 9 g gDNA to remove any contaminating rRNA and host DNA before the enzymatic degradation. Figure 1 shows the nucleoside composition profile of 9 g gDNA with an estimated dG^+^/dG ratio of 25–27%, which is in the same range as previously reported^14^.

### Restriction digestions of phage 9 g gDNA by Type II REases

**Figure 2:**
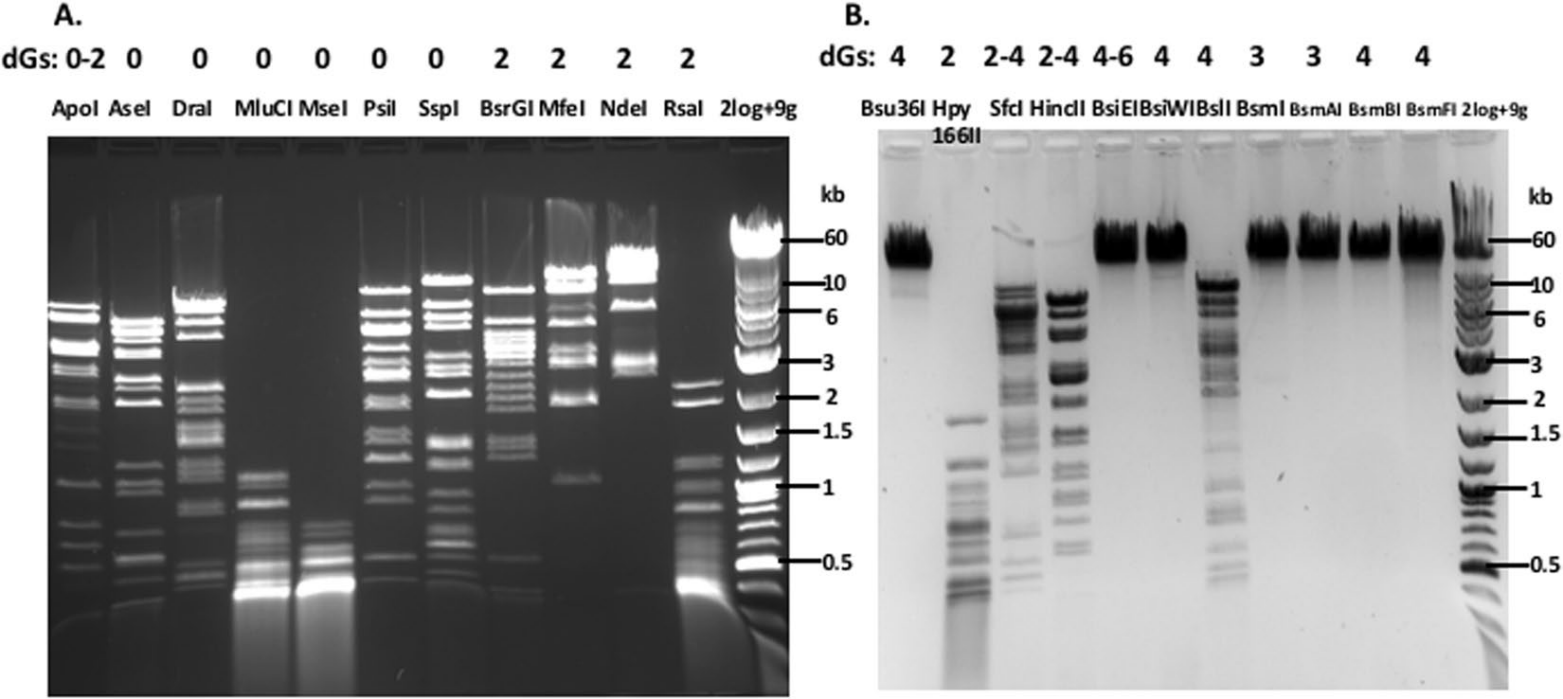
Restriction digestion of phage 9 g DNA by Type II REases. (**A**) Restriction digestion of phage 9 g DNA by Type II REases with A/T only or A/T rich recognition sequences. REases were indicated on top of each lane as well as the number of guanine bases (G) in the recognition sequence. The exact number of dG^+^ bases in a particular restriction site is unknown. Refer to computer-generated restriction patterns in the Supplementary Material for reference. (**B**) Restriction digestion of phage 9 g DNA by Type II REases and analysis of restricted fragments on 1% agarose gels.

Since the major modified base in phage 9 g is the guanine derivative dG^+^, it is anticipated that REases displaying all A/T bp in their recognition sequence﻿s﻿ should cleave the substrate DNA efficiently. Figure 2A shows that AseI (ATTAAT), DraI (TTTAAA), MluCI (AATT), MseI (TTAA), PsiI (TTATAA), and SspI (AATATT) indeed cleaved the gDNA efficiently resulting in complete digestions. PacI (TTAATTAA) also digested the phage DNA to completion as confirmed by multiple double digestions (data not shown). Unlike the α-putrescinylthymine modification in φW-14, which has neighboring inhibitory effects on the restriction of some G/C only sites (SYX, unpublished results)^10^, the dG^+^ modification appears to have a minimal inhibitory effect on the restriction of A/T only sites. There were a few differences in the predicted and actual banding patterns which were attributed to the disagreement of the linear DNA deposited in GenBank and the actual physical ends generated by phage terminase during DNA packaging and phage maturation (see below). Four REases with A/T rich recognition sequences, ApoI (R↓AATTY), BsrGI (T↓GTACA), MfeI (C↓AATTG), and NdeI (CA↓TATG), digested the substrate partially (Fig. 2A). RsaI (GT↓AC) appeared to digest 9 g gDNA to completion even though its recognition sequence contains two guanines in opposite strands near the cleavage sites. Conversely, phage 9 g gDNA was completely resistant to BamHI, whose recognition sequence G↓GATCC contains four guanines directly at the cleavage sites of two opposite strands. Analogous resistance to cleavage was observed for EcoRI and HindIII, whose recognition sequences G↓AATTC and A↓AGCTT contain two symmetrical guanines in opposite strands (data not shown). The computer-generated digestion patterns by NEBcutter in Supplementary Figure 1 provide a reference to the extent of the restriction digestions. It is not clear whether the failure of some REases to cleave phage 9 g DNA occurred in the DNA binding step (transition from non-specific binding/sliding to specific binding) or in the catalytic step.

We carried out restriction digestions for all Type II REases listed in the NEB’s catalog (see Supplementary Table 1). Selected results are presented in Fig. 2B. BslI (CCN_7_GG), HincII (GTYRAC), and SfcI (CTRYAG) digested phage 9 g DNA partially. Bsu36I, BsiEI, BsiWI, BsmI, BamAI, BsmBI, and BsmFI digested phage 9 g DNA poorly. Hpy166II (GTN↓NAC) was able to digest the DNA to completion despite the presence of two guanines in the recognition sequence, probably due to the cleavage site located within the non-specific bases. TaqI (TCGA) endonuclease, a thermophilic enzyme also digested the DNA into completion (data not shown). We also carried out restriction digestions for Type IIM REases DpnI (G^N6m^ATC) and MspJI (^5m^CNNR), Type III REase EcoP15I (CAGCAG, + ATP), Type IV REase McrBC (Pu^5m^C-N_40-3000_-Pu^5m^C), and the modification-dependent enzyme BisI (G^5m^CNGC, 2–4 ^5m^C, wherein the cytosine opposite to the G is modified ^5m^C). The digestion results are shown in Supplementary Table 1. We divided all tested REases into four major groups: complete digestion (c), partial digestion (p), very partial digestion (vp, majority of the substrate DNA stays intact, only a few weak digested products visible), and resistant (x). All complete and partial digestions were repeated to confirm the positive results. Overall, 29% of the REases digested phage 9 g DNA completely or partially (~14.5% enzymes achieved complete digestions). The dG^+^-modified DNA was mostly resistant to the remaining 71% of REases. The modification-dependent REases BisI, MspJI, and McrBC failed to digest phage 9 g DNA due to the lack of ^m5^C modification (as controls BisI, MspJI, and McrBC were able to cleave ^m5^C-containing phage XP12 DNA). For 6, 7, and 8 base cutters, if the recognition sequence contains more than three guanines, the site in phage 9 g DNA is most likely resistant to Type II restrictions. But the resistant sites appeared to lack a specific pattern. For example, when the flanking sequence X_1_ and X_2_ in X_1_ GX_2_ of all resistant sites were compiled by Weblogo (http://weblogo.berkeley.edu/logo.cgi), no particular base bias (specificity) appeared to be detected in the X_1_ and X_2_ positions (data not shown). For frequent cutters (3–5 bp) or REases with degenerate recognition sequence, e.g., HincII (GTYRAC, partial) and NspI (RCATGY, partial), the possibility of complete or partial restriction was less predictable. Interestingly, phage 9 g DNA is resistant to all six Type II REases (Eco53kI, EcoNI, EcoO109I, EcoP15I, EcoRI, EcoRV) isolated from *E*. *coli* strains. In summary, dG^+^-modified bases in phage 9 g DNA rendered the genome resistant or largely resistant to ~71% of Type II restrictions *in vitro*. This is quite remarkable since only 25% to 27% of guanines are replaced by dG^+^ in its genome.

As a control experiment, we performed digestion of λ DNA by 20 REases that failed to digest 9 g DNA. We confirmed that those 20 enzymes were active and able to cleave λ DNA (data not shown). For infrequent cutters, we also performed computer-generated restriction digestion to check the existence of restriction sites. Another precaution was to use fresh batches of REases that had recently gone through rigorous quality control by the manufacturer. By taking these precautions, we expected to minimize the number of false negatives in our restriction assays. We found one potential false negative from a previously published result on the restriction of phage 9 g DNA. DraI (TTTAAA) was reported not able to cleave the substrate DNA^13^. In our hands, DraI is fully active in restricting phage 9 g DNA. DraI from two other suppliers is also able to cleave the DNA (see Supplementary Material).

We also tested a few Type IIS REases with asymmetric recognition sequences that contain guanines on only one strand (the G-strand), while the opposite strand contains the complementary cytosines (the C-strand). For example, BseRI recognition sequence is 5′GAGGAG3′ (G-strand) and the complementary strand is 5′CTCCTC3′ (C-strand). EarI target site contains 5′CTCTTC3′ (C-strand) and the opposite strand 5′GAAGAG3′ (G-strand). Phage 9 g DNA was resistant to BseRI and EarI digestion (data not shown); however, it was not clear whether the C-strand was actually cleaved or nicked since the digested DNA was analyzed by agarose gel electrophoresis. To find out the answer, we performed DNA sequencing reactions following BseRI and EarI digestions using primers designed in close proximity to the BseRI and EarI sites. No run-off sequence (a sudden sequencing peak drop off resulting from broken template) was detected, indicating that BseRI and EarI did not cleave/nick the C-strand (data not shown). This result suggests that the incorporation of dG^+^ into only one strand (the G-strand) can block Type IIS restriction (in particular 6–8 bp G/C rich recognition sequences).

### Ligation of phage 9 g DNA fragments with sticky or blunt ends

**Figure 3:**
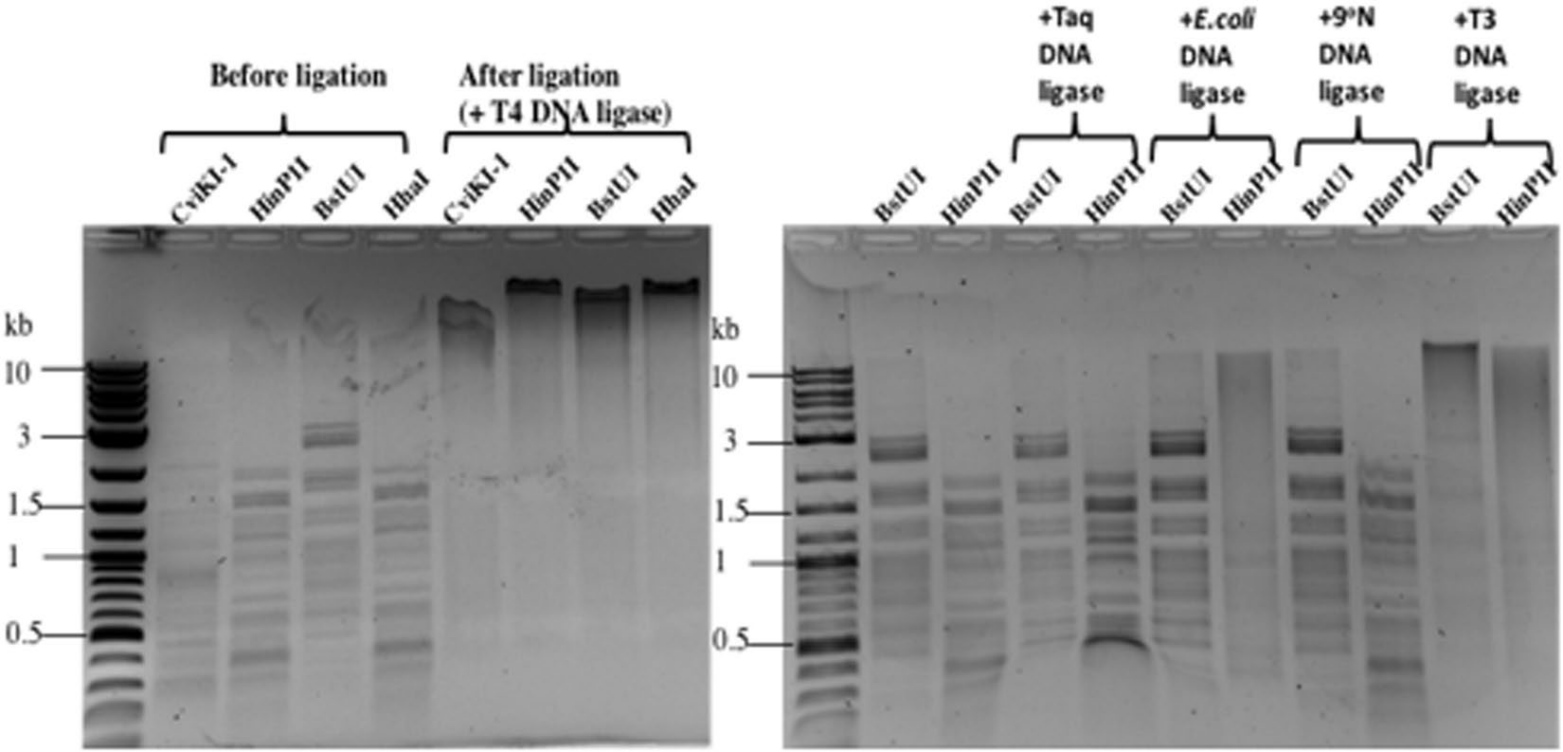
Ligation of phage 9 g restriction fragments by T4, T3, and *E*. *coli* DNA ligases.

We examined the ligation efficiency of restriction fragments derived from the restriction digestion of phage 9 g gDNA. Four REases that cleaved phage 9 g DNA partially were tested: BstUI (CG↓CG) and CviKI-1 (RG↓CY), which generate blunt ends; and HhaI (GCG↓C, 2-nt 3′overhang) and HinP1I (G↓CGC, 2-nt 5′-overhang), which create sticky ends. Following restriction digestion and spin column purification, the restriction fragments were subjected to ligation by T4 DNA ligase. All four restriction fragments were ligated into larger products after 4 h ligation at room temperature (Fig. 3, left panel). The BstUI and HinP1I fragments were also ligated by T3 DNA ligase (Fig. 3, right panel). *E*. *coli* DNA ligase was able to ligate the HinP1I restriction fragments with two-base overhang, but it ligated the BstUI blunt fragments poorly. It is known that *E*. *coli* DNA ligase prefers to ligate duplex DNA containing cohesive ends. Taq and 9°N DNA ligase failed to ligate BstUI and HinP1I fragments after 4 h incubation at 45 °C or 60 °C, which is consistent with the fact that Taq and 9°N DNA ligases prefer to ligate nicked duplex DNA and seal the nicks with 5′-phosphate and 3′-hydroxyl termini. In summary, T4 and T3 DNA ligases were active in the ligation of phage 9 g restriction fragments regardless of whether they contained blunt or cohesive ends. *E*. *coli* DNA ligase was capable of ligating phage 9 g fragments with sticky ends. Taq and 9°N DNA ligases appeared to ligate phage 9 g restriction fragments poorly at 45 °C or 60 °C. We have not tested phage 9 g DNA ligase since this enzyme has not been purified.

### Exonuclease digestion of SspI fragments of phage 9 g DNA

The SspI restriction fragments from phage 9 g DNA were degraded by BAL31 nuclease, phage λ exonuclease, phage T5 exonuclease, T7 exonuclease, and *E*. *coli* exonuclease V (data not shown). In control experiments, SspI restriction fragments from phage λ DNA had a similar exonuclease digestion profile (data not shown). In summary, we did not observe obvious difference in the exonuclease degradation of dG^+^-containing DNA restriction fragments compared to regular DNA.

### Modification of phage 9 g DNA by MTases

**Figure 4:**
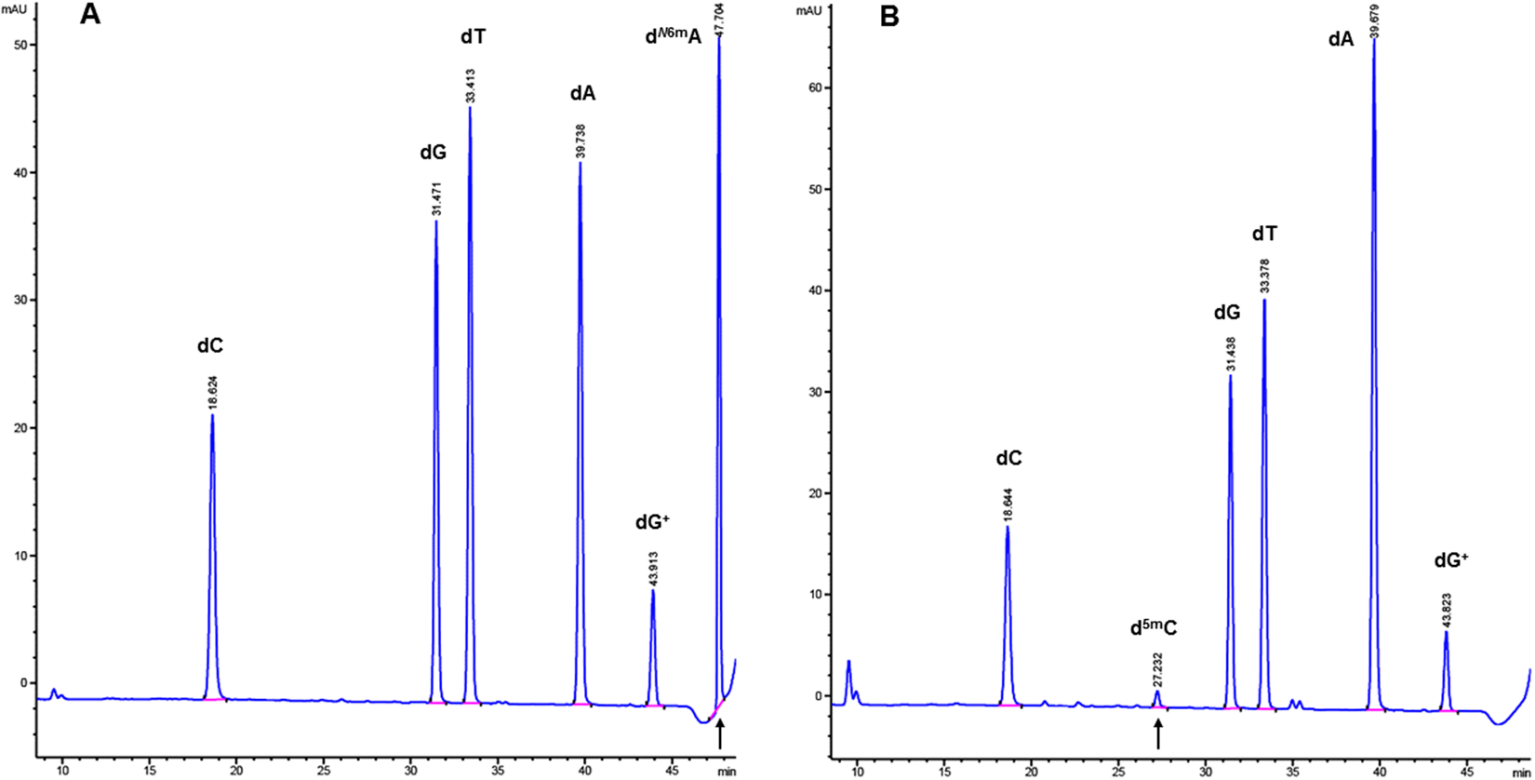
LC-MS analysis of (**A**) M.EcoGII (frequent adenine MTase to yield ^N6m^A) or (**B**) M.CviPI (GpC MTase to yield ^5m^C)-modified phage 9 g DNA.

**Figure 5:**
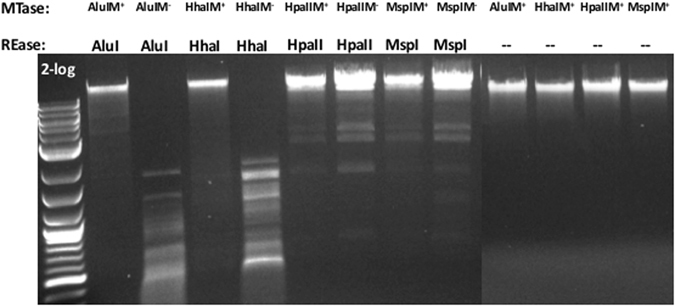
Modification of phage 9 g DNA by DNA MTases and restriction of the modified DNA by the cognate REase. The modified DNA samples (unrestricted, lanes 10–13) were run on the same gel, but on a different row and imaged separately.

We examined the possibility of modifying phage 9 g DNA with the frequent adenine MTase EcoGII (predicted site preference AB, wherein B=C, G or T) or the GpC MTase (M.CviPI). After the methylation reaction, the modified DNA was gel-purified and subjected to non-specific nuclease degradation and dephosphorylation to mononucleosides, followed by LC-MS analysis. Approximately 45% of the adenines were converted to ^*N*6m^A after a two-hour treatment with EcoGII (Fig. 4, left panel), while the dG^+^ composition remained unchanged at 25%. Only 7% of the cytosines were converted to ^5m^C after treatment with M.CviPI under similar condition suggesting that dG^+^-containing DNA is not as a good substrate for M.CviPI. We also attempted to modify phage 9 g DNA with M.AluI (AGCT to AG^5m^CT), M.HhaI (GCGC to G^5m^CGC), M.HpaII (CCGG to C^5m^CGG), and M.MspI (CCGG to^5m^CCGG). After the methylation reaction, the phage DNA was purified by spin column and digested by the corresponding cognate REase. Following modification by M.AluI and M.HhaI, the 9 g DNA became mostly resistant to AluI and HhaI restriction, respectively (Fig. 5). But M.HpaII and M.MspI failed to modify phage 9 g DNA, since the MTase-treated DNA gave rise to similar partial digestion pattern as the unmethylated DNA (note: HpaII and MspI only cleaved phage 9 g DNA partially). In summary, phage 9 g DNA can be further modified by frequent adenine MTase EcoGII and cytosine MTases M.AluI and M.HhaI, and to a lesser extent by M.CviPI.

### The use of phage 9 g DNA as template in PCR

We examined the use of phage 9 g DNA as a template in PCR using thermostable DNA polymerases. Eight sets of PCR primers were synthesized based on phage 9 g coding sequences for primase, DNA ligase, DNA polymerase (two subunits/ORFs), and five predicted genes involved in the dG^+^ synthesis pathway, namely GTP cyclohydrolase (GCYH), 6-pyruvoyl-tetrahydropterin synthase (PTPS), glutamine amidotransferase (GAT)-QueC enzyme, QueE-like radical activating enzyme, and archaeosine-tRNA-ribosyltransferase (aTGT, a.k.a. tRNA guanosine transglycosylase). Eight PCR reactions were carried out using Phusion DNA Polymerase. PCR products were detected in all eight PCR reactions (data not shown). In another experiment, a set of primers was used to amplify the coding sequence for GAT-QueC gene using Phusion, Q5, and Taq DNA Polymerases. Phusion and Q5 DNA Polymerases produced PCR product in high yield, while the yield with Taq DNA Polymerase was poor. Increasing Mg^2+^ concentration to 4–6 mM and PCR cycles from 25 to 30 greatly improved the product yield of Taq DNA Polymerase (data not shown).

### Restriction of phage 9 g (phage spot test)

Phage 9 g was originally isolated from horse feces and plated on *E*. *coli* K12 strain C600^13^. We used 9 g DNA to transfect *E*. *coli* K strains NEB Turbo and 10β. Phage 9 g was able to form plaques on both plating hosts, each of which lacked the EcoK12 Type I restriction system and ^5m^C-dependent restriction system McrBC. Although NEB Turbo carries the McrA and Mrr restriction systems, it is permissive to phage 9 g (McrA restricts/binds ^m5^CGR sites and Mrr restricts ^5m^C and ^*N*6m^A modified DNA)^23, 24^.

To further test restriction of phage 9 g, we used a number of WT *E*. *coli* strains collected from the environment (avian and mammal sources). Phage 9 g stock was diluted and spotted on cell lawns of WT and two lab strains NEB Turbo and 10β. Phage 9 g formed clear spots (indication of cell lysis) on NEB Turbo and 10β, but it did not form clear plaques (spot) on the seven WT hosts tested. However, phage 9 g formed cell inhibitory zones with undiluted phage stock (data not shown), implicating phage 9 g absorption and disrupted cell membrane function at high phage titers in some of the WT strains. Table 1 shows the *in vivo* phage restriction activity of two *E*. *coli* lab strains and seven WT strains. In control experiment, phage λ and T4gt (^5hm^C) were mostly restricted by these seven WT strains. Four WT strains were able to restrict the hypermodified phage T4 with ^glc-5hm^C. Further study is required to understand the phage attenuation mechanism.

**Table 1:** Phage plaque formation (phage spot test) on two *E*. *coli* lab strains and seven WT *E*. *coli* isolates.

	Phage 9 g (dG^+^)	Phage λ (normal dG)	Phage T4gt (^5hm^C)	Phage T4 (^glc-5hm^C)
***E***. ***coli*** **host**				
NEB 10β	+	+	+	+
NEB Turbo	+	+	−	+
MB3565	−	−	−	+
MB3646	+/−	−	−	−
MB3647	+/−	−	+/−	+
MB3648	+/−	−	−	+
MB3649	+/−	−	−	−
MB3650	+/−	−	−	+/−
MB3651	+/−	−	−	+/−

+, Forming plaques or cell inhibitory zone (circle). −﻿, No plaques (i.e., phage restricted or attenuated). +/−, forming cell inhibitory zone only at the high phage titer (i.e. undiluted phage stock). NEB Turbo (Mrr^+^ and McrA^+^) restricted ^5hm^C-containing T4gt. MB3565 is a WT *E*. *coli* isolate from an avian source. MB3646 to MB3651 are WT *E*. *coli* isolates from mammals.

### Digestion of phage 9 g and λ DNA WT *E. coli* cell extracts

We envision that some WT *E*. *coli* hosts may have evolved modification-dependent Type IV REase that can specifically attack dG^+^ modified DNA, analogous to *E*. *coli*-encoded GmrSD endonuclease cleavage of T4 and T4gt DNA. If such enzymes existed in nature they would presumably cleave phage 9 g DNA more effectively than λ DNA. Cell extracts were prepared from 22 WT *E*. *coli* strains, and used to digest full-length phage 9 g and λ BstEII restriction fragments *in vitro*. Supplementary Figure 2 shows the digestion results of two mixed DNA substrates. Nucleases from eight cell extracts apparently cleaved phage 9 g and λ DNA into smaller fragments and caused extensive smearing (~36% positive). One cell extract (MB3634) cleaved λ DNA faster than phage 9 g, implying this strain probably contains a Type II REase that cleaves λ DNA, but not phage 9 g. Another extract (MB3635) appeared to digest phage 9 g faster than λ DNA. It is not clear whether the nuclease activity was due to Type I, II, IIM, III, or IV REase, or simply non-specific endonuclease and exonuclease. Further extensive enzyme purification is required to sort out the type of endonuclease and exonuclease that preferentially cleave the phage 9 g DNA.

### DNA endonuclease and exonuclease associated with TGT

It has been shown that the *dpd* gene cluster of *S*. *enterica* serovar Montevideo encodes a REase that restricted unmodified plasmid DNA by over 1,000-fold^14^. Thus the *dpd* gene cluster encodes a novel R-M system (a PLD-family endonuclease (DpdK), a DNA helicase (DpdJ), a possible specificity subunit, and dG^+^ modification enzymes). To uncover other DNA endonuclease and exonucleases associated with the *tgt* gene (aTGT-like or bTGT-like) in other bacterial/archaea genomes we used the SEED Viewer Server to examine nearby genes. We found additional DNA metabolic enzymes such as DNA integrase, transposase, DNA helicase and PD-D/EXK nuclease (Cas4-like exonuclease), DNA MTase, resolvase, and toxin-antitoxin pair adjacent to the *tgt* gene (data not shown). Phage 9 g encodes a Cas4-like exonuclease and a DNA helicase next to the dG^+^ modification genes^14^, a similar arrangement found in *M*. *aeolicus* and *D*. *thermolithotrophum* genomes. We also used the bacterial QueC (7-cyano-7-deazaguanine synthase) as a “seed” to search for DNA endonucleases and exonucleases located in close proximity in the genomes. Supplementary Figure 3 shows seven genomic loci with a QueC-GTP cyclohydrolase-tRNA guanosine transglycosylase gene cluster. The predicted DNA metabolic enzymes include a DNA primase and a Type I R-M system in two strains of *Geobacter uraniireducens*, a PLD-family protein in *Geobacter metallireducens* and *Geobacter sulfurreducens*, and homologs of DNA endonuclease III, UvrABC exinuclease, and transposase in three other bacterial strains. In the two strains of *G*. *uraniireduces*, four ORFs (hypothetical proteins including a putative Zn-dependent hydrolase (amino deaminase﻿?﻿) were inserted between the *hsdM-hsdS* and *hsdR* genes of a Type I R-M system. One of the four ORFs is a Piwi-domain protein that may encode a RNA-guided interference system^25^. In summary, by searching for a limited number of annotated genomes in SEED Viewer Server, one example of Type I R-M system, a metal-dependent hydrolase, and possibly a RNA-guided RNA interference system were closely associated with preQ_0_ biosynthesis gene cluster in two bacterial strains. The rest of DNA/RNA metabolic enzymes seem to lack a consensus functionality and physical co-localization in regards to aTGT/bTGT or QueC.

### Isolation of phage 9 g resistant NEB 10β mutants

The *E*. *coli* outer membrane receptor for phage 9 g absorption and DNA transport is unknown. Phage 9 g is a lytic phage that does not appear to encode its own integrase for integration into the host chromosome to form a lysogen. During phage 9 g plating we often observed phage resistant cells (colonies) that outgrew from a lysed cell lawn following incubation for a few days at room temperature, presumably as the result of spontaneous mutations (Supplementary Figure 4A). Four of these outgrowth colonies were streaked out twice to form single colonies. Cell lawns of the phage resistant colonies were spotted with phage 9 g, λ, and T4. As expected, the three phages were able to form plaques (lysed cell spot) on the parent NEB 10β cells (Supplementary Figure 4B). Phage 9 g and λ were unable to form plaques or cell inhibitory zone on all four mutants, although the phage 9 g resistant cells were permissive to phage T4 (Supplementary Figure 4C and D, data not shown for other two mutants). This result implies that Phage 9 g and λ may share the same host receptor LamB for phage tail absorption and DNA delivery (LamB also serves as bacterial maltose/maltodextrin transport pore in the outer membrane)^26^, whereas phage T4 utilizes the host lipopolysaccharide and outer membrane protein OmpC for phage absorption and DNA injection^27^. Further sequencing of the *lamB* (*malB*) gene and the genes in the maltose operon would be required to pinpoint the exact mutation(s). In summary, phage 9 g resistant NEB 10β mutants can be readily isolated in phage plating. Phage 9 g and λ are likely to share the same host receptor (LamB) for phage absorption and DNA injection/transport.

### Possible dG^+^ modification in bacteriophage and bacterial genomes

The 7-cyano-7-deazaG synthase is a bifunctional enzyme containing the amidotransferase at the *N*-terminal (GnAT II superfamily) and QueC at the *C*-terminal domain. We used the amino acid sequence of 7-cyano-7-deazaG synthase of phage 9 g to search for similar proteins in GenBank and found five close homologs from two *Pseudomonas* phages (NP1 and Pamx25, 52% aa sequence identity) and three *Enterobacteria* phages (JenK1, JenP1, and JenP2, with 95% to 98% aa sequence identity)^16^ (Supplementary Figure 5). The gene clusters also encode other enzymes required for dG^+^ synthesis and incorporation into the phage genome. It has been postulated that the *Pseudomonas* phages are hypermodified because the gDNA is resistant to many Type II restrictions^15^. A large number of homologs of 7-cyano-7-deazaguanine synthase were found in archaea that share moderate sequence similarity to the phage encoded enzyme (data not shown).

It is known that some bacteria possess the preQ_0_ (7-cyano-7-deazaG) and preQ. _1_ (7-aminomethyl-7-deazaG) synthesis pathway that produces 7-deazaG derivative queuosine (Q) for bacterial tRNA modification^28, 29^. In some archaea, the preQ_0_-tRNA is further modified to become archaeosine-tRNA. Thus both tRNA and DNA modifications share the key intermediate preQ_0_. The enzyme glutamine amidotranferase-QueC catalyzes the conversion of carboxydeazaG (CDG) to 7-amindo-7-deazaG (ADG) and preQ_0_. In archaea, preQ_0_ is the substrate for aTGT^28^. PreQ_0_ and archaeosine nucleoside have been synthesized chemically^30^. A four-step enzymatic reactions to synthesize preQ_0_ from guanine *in vitro* has been reported previously^31^.

### Mapping terminase cleavage site (*pac* site) of phage 9 g by direct sequencing of the phage DNA

**Figure 6:**
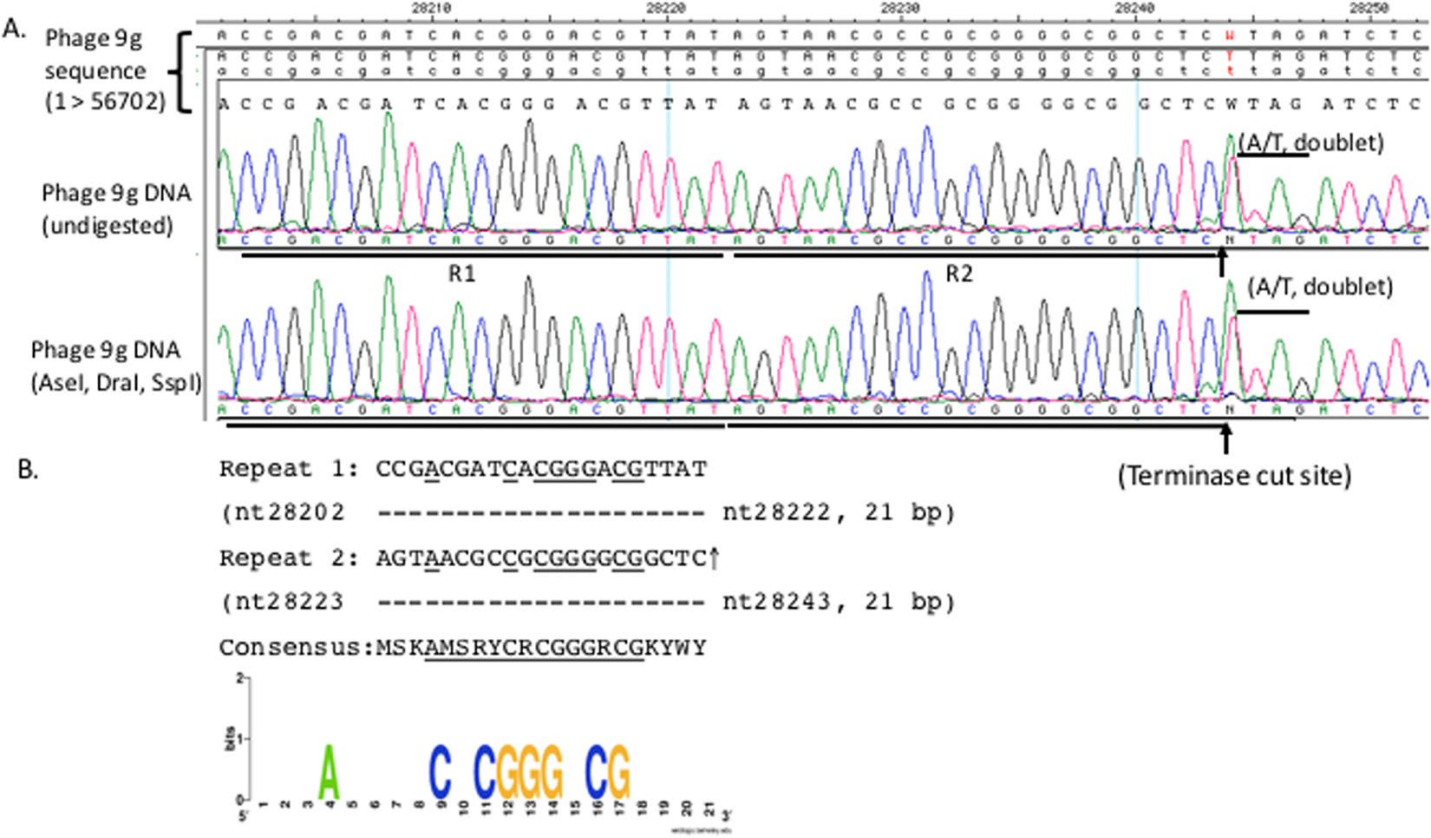
Direct DNA run-off sequencing using phage 9 g genomic DNA as template. (**A**) DNA sequencing peaks drop off after nt 28244, and double peaks at nt 28243 (A/C) and nt 28244 (A/T). Note: the template-independent extra A peak in the doublet is slightly higher than the T peak at nt position 28244 due to a major bottom-strand cut at nt 28243. Upward arrows indicate the bottom strand is cleaved (nicked). (**B**) Two 21-bp direct repeats adjacent to the terminase cut site. A core consensus sequence of 12 bp (SRYCRCGGGRCG) can be found in the two sequences. The Weblogo server was used to create the consensus sequence. A third copy of the 21-bp direct repeat is located in the *tgt* gene (not shown here).

Since phage *cosB*/*cosN* (cohesive end site for terminase binding/nicking) and *pac* (packaging) sites are physically located in close proximity to terminase genes we used primer walking to directly sequence the phage genomic region upstream of the terminase B (*terB*) gene at nt 25175 to nt 32833 (~7.7 kb in length). Figure 6A shows the sequencing peaks near the terminase cleavage site (*pac* site). There is a doublet peak (W = A/T) at nt position 28244 and a﻿﻿ sudden drop of peak height at nt 28245, indicating the broken DNA templates at nt 28243 (where it had been cut by the phage terminase). This major terminase cut site in the genomic DNA was estimated between 33% to 50% of all cuts made to the bottom strand (the template-independent extra A peak is slightly higher than the overlapping T peak), based on the control run-off peaks of mixed DNA templates with cut and uncut DNA molecules (see Supplementary Figure 6). In the shot-gun sequencing of phage 9 g DNA fragments in a NGS library, there were high occurrences (over 100 reads) starting or ending with this position (nt 28243), implying a natural cut introduced by the terminase during phage DNA packaging^13^. Two 21-bp direct repeats with a 12-bp core sequence (SRYCRCGGGRCG, S = C/G, Y = C/Y, R = A/G) precede the major bottom-strand cut site (Fig. 6B). A minor cut (bottom-strand) was also detected at nt position 28242 (a C/A doublet) possibly due to the removal of one base by *E*. *coli* exonuclease during phage DNA preparation or imprecise cut by the terminase. Supplementary Figure 7A–C show a schematic diagram of cleavage at the *pac* site and the possible DNA packaging direction. Based on the viral DNA run-off sequencing data, we propose that phage 9 g terminase makes a major cut at nt 28243 and initiates DNA packaging leftward (i.e. nt 28243 is the first base to be packaged, being the number 1 base of the physical DNA end) (Supplementary Figure 7B,C). When the terminase encounters a second *pac* site, it does not make any cleavage. Instead, it uses a “headful” packaging mechanism to terminate the DNA translocation process after passing the second *pac* site, thus creating a terminal-end redundant sequence (see below)^32, 33^. A cryptic *pac* site (12-bp core sequence SRYCRCGGGRCG) is found in the phage *tgt* gene with unknown function (data not shown). Inspection of *Enterobacteria* phage JenK1, JenP1, and JenP2 showed that these three phage carry the same *pac* sequence in their genomes and highly homologous terminase coding sequences (data not shown)^16^. The exact nature of the terminase cut ends (blunt ends or staggered ends) remain to be investigated in future study.

During DNA sequencing and identification of the potential *pac* site, we also found ten differences in base calling compared to the published genome sequence (nt 25175 to nt 32833, ~7659 bp)^13^. They are listed in Supplementary Table 2. The difference could be result of sequencing/editing errors or variations in the phage DNA after many rounds of passage under laboratory conditions.

### DraI digestion of the *pac* DNA fragment

The DraI REase (TTTAAA) cleaves phage 9 g into >20 fragments (Supplementary Figure 8). The computer generated fragments (NEBcutter) are shown in Supplementary Figure 8B and C. There are two major differences in the actual and predicted restriction patterns. #9 (1754 bp, left end) and #24 (440 bp, right end) fragments appeared in a single fragment of 2.2 kb (indicated by arrow 3). Direct DNA sequencing using phage 9 g DNA as templates also shows that this region is 100% continuous and is not the physical ends of the viral genome (data not shown). The predicted 13.5 kb DraI fragment encompassing the *pac* site was missing. But two overlapping fragments (a doublet) of ~8.4 kb were detected in the agarose gel as the result of terminase cut at the *pac* site (indicated by arrow 2). Terminase cleavage of the 13.5 kb fragment would have generated two fragments of predicted length of 8.4 kb and 5.2 kb (however, the 5.2 kb fragment is missing in the gel). To reconcile the size difference, we propose that the phage DNA ends may contain approximately 3.0 to 3.2 kb terminal redundant repeat with circular permutation. This terminal repeat would package ~5.6% of extra length of DNA into the prohead as the result of “headful” packaging and non-specific cleavage after the second or third *pac* site. DraI REase from two other suppliers produced a similar digestion results, except a slow migrating band that might be caused by DNA binding proteins in the enzyme preparation (indicated by arrow 1). Alternatively, the extra weak band might be caused by artifact of gel electrophoresis.

To further confirm the cleaved *pac* site in the viral DNA (termianse cut at nt 28243) we performed restriction of 9 g DNA with AseI, MluCI, PsiI, RsaI, and SspI, respectively. The *pac* fragments cleaved by the phage terminase and restriction enzyme were clearly identified (Supplementary Figure 9, indicated by “*”). For example, the predicted PsiI-*pac* fragment (nt 25215 to 28243 = 3028 bp) and the RsaI-*pac* fragment (nt 26291 to 28243 = 1952 bp) were clearly detected in the agarose gel. In summary, direct DNA run-off sequencing from the genomic DNA and restriction mapping of the *pac* fragments confirmed the terminase cut site and the major broken point previously reported for the phage DNA^13^.

A BglII site (AGATCT) is 2 bp away from the major terminase cut site, but the phage DNA is resistant to BglII digestion (see resistant sites in Supplementary Table 1). BstYI with degenerate recognition sequence (RGATCY) can partially digest phage 9 g DNA at 60 °C. But its cleavage efficiency near the *pac* site warrants further study. To study the *pac* site region we recommend using REases with 4–6 bp A/T only or A/T rich target sites (Supplementary Material).

## Discussion

### Phage 9 g DNA sensitivity to Type II restriction

Phage 9 g DNA can be digested completely or partially by ~29% of Type II REases examined in this work. The hypermodified DNA is resistant (or mostly resistant) to ~71% of Type II restrictions. But no apparent sequence motif (specificity) was found among the resistant sites. Further chemical or enzymatic modification of the dG^+^ base in phage 9 g DNA is required for direct detection of the modified base to determine any sequence specificity. We have not tested phage 9 g DNA sensitivity to Type I restriction systems, which consist of two bipartite recognition sequences (rs) (TRD1rs-Nx-TRD2rs, TRD1rs + TRD2rs = 5–9 bp). We anticipate that phage 9 g DNA would be restricted if the Type I specificity contains A/T only rs or partially restricted, if the restriction sites contains A/T rich sequences, for example, Btr188III (ACAN_6_TTTA), Eco2747AII (GAAN_7_TAAA), and Hin8143I (TTTAN_7_GTT) in analogy to the Type II restriction systems tested above. Among the known Type I specificities listed in REBASE, A/T only recognition sequences of TRD1 or TRD2 have been found. However, Type I specificities with A/T only sequences of both TRD1﻿rs and TRD2﻿rs have not been reported^34^. This observation suggests that phage 9 g DNA is likely resistant to Type I restrictions.

Another strategy for phage to combat Type II restriction is to avoid such restriction sites in its genome. Phage 9 g DNA does not carry the recognition sequence for 13 six-base cutters (Acc65I, AfeI, ApaLI, AvrII, BmtI, BssHII, HindIII, KpnI, NheI, ScaI, SnaBI, SpeI, and SphI). Why these 6-base restriction sites were absent is not clear or the lack of these restriction sites could be just coincidence. The phage genome also lacks RsrII sites (a 7-base cutter) and four restriction sites for 8-base cutters due to the less frequent occurrence in the genome.

### Ligation of phage 9 g DNA fragments

In the limited number of restriction fragments tested in this work, phage T3 and T4 DNA ligases were able to ligate phage 9 g DNA fragments with blunt or sticky ends. Phage 9 g encodes its own DNA ligase, primase, and DNA polymerase in the genome for phage DNA replication. Presumably, the phage 9 g DNA ligase has evolved to efficiently ligate the dG^+^-containing Okazaki fragments during lagging strand synthesis.

### Phage 9g-encoded nuclease and DNA helicase

Phage 9 g genome encodes a Cas4-like exonuclease and an adjacent DNA helicase in close proximity to the dG^+^ synthesis genes. The function of the phage encoded nuclease and helicase is still unknown. They may be part of a restriction system that restricts unmodified DNA (or degradation of host DNA for mononucleotide recycling), analogous to the restriction system encoded by the *tgtA5* gene cluster^14^.

### Additional modification of phage 9 g DNA

Phage 9 g DNA can be further modified by frequent adenine MTase EcoGII and cytosine MTases M.AluI and M.HhaI, and to a lesser extent by M.CviPI. These results indicate that ^5m^C or ^*N*6m^A can be incorporated into phage 9 g DNA *in vitro*, besides the presence of the modified base dG^+^.

For preparation of hypermodified phage 9 g DNA (dG^+^ and ^5m^C and/or ^*N*6m^A), we envision cloning *ecoGIIM*, *aluIM*, or *hhaIM* genes into phage 9 g genome under the phage early gene promoter for improved expression and modification. The phage 9 g DNA with double or triple modified bases might be more efficient in killing some pathogenic *E*. *coli* strains (assuming they lack dG^+^-dependent restriction system).

### Restriction of phage 9 g by WT *E. coli* strains and digestion of phage 9 g by WT cell extracts

We found a panel of WT *E*. *coli* strains restricted phage 9 g. The non-permissive hosts may contain: (1) inhibition of phage adsorption or lack of phage receptor due to mutation, (2) restriction systems (DNA endonuclease, RNA-guided DNA/RNA endonuclease, e.g. CRISPR-Cas acquired immunity systems that restricted phage replication^35^, (2) DNA glycosylase/AP lyase that can remove the modified base to create AP sites and multiple nicks in phage DNA, (3) DNA exonucleases that cause extensive damage to the ends of phage genome, (4) RNA endonucleases (e.g. tRNase that destroys dG^+^-carrying tRNA), (5) Proteinase to target phage morphogenesis proteins, (6) certain lysogenic phage encoded proteins that interferes with phage superinfection similar to the superinfection exclusion system (Sie)^36^, or other unknown phage attenuation mechanisms that inhibit phage growth.

Among the 22 WT *E*. *coli* cell extracts tested, about one third of extracts degraded both phage 9 g and λ BstEII DNA possibly by Type II REases and exonucleases. It is interesting to see that some WT cell extracts did not effectively cleave phage 9 g and λ DNA *in vitro*, but these strains were able to inhibit phage 9 g growth *in vivo*. In theory, cloning and expression of the inhibitory factor(s) into a permissive host (i.e. NEB Turbo) would render the new host resistant to phage 9 g infection.

### Modification-dependent restriction systems

Among the 22 WT *E*. *coli* cell extracts examined for endonuclease activities, only one cell extract appeared to degrade phage 9 g DNA more extensively than λ DNA (see Supplementary Figure 2). But further enzyme purification and substrate characterization are required to pinpoint the possible modification-dependent endonuclease activity. The modification-dependent REases have been classified as Type IIM with defined cut sites (e.g. DpnI-G^*N*6m^ATC, MspJI-^5m^CNNR, NhoI-G^5m^CNG^5m^C, and Esp638I-G^5m^CNNG^5m^C, PvuRts1I-^5hm^C-N_22_-G) or Type IV with undefined cut sites (*E*. *coli* McrBC-Pu^5m^C-N_40-3000_-Pu^5m^C, *E*. *coli* GmrSD-^5hm^C-N_(17-23)_-G, SauUSI-S^5hm^CNGS). In the restriction enzyme data base (REBASE)(www.rebase.neb.com) there are 517 characterized and putative Type IIM REases from bacteria^34^. Most of them belong to the BisI family^37^, MspJI family^38^, DpnI family^39^, and PvuRts1I family^20^ of enzymes. Some BisI homologs with low sequence similarities have diverged into new modification-dependent endonuclease specificities (SYX, unpublished results). Over 9200 putative Type IV REases are listed in REBASE, most of which are GmrSD, McrBC, Mrr, and SauUSI homologs found in bacteria^34^. Another interesting type of DNA modification is the DNA backbone phosphorothioate modification, which is targeted by a modification-dependent endonuclease ScoMcrA that shows low sequence homology to the HNH nuclease domain of EcoMcrA^22^. The discovery and characterization of these modification-dependent REases will facilitate the utilization of these enzymes in epigenetics, cancer research, and in recombinant DNA technology. Another group of bacterial enzymes contains the N-terminal Sra domain (binding to hemi-methylated and fully methylated ^5m^C DNA) and the C-termianl HNH nuclease domain. Two Sra-HNH proteins have been found to tightly bind ^5m^C-DNA^40^, similar to McrA protein that binds to ^5m^CGR^41^, but they display limited non-specific endonuclease activity. Further genome mining work from sequenced microbial genomes may uncover more modification-dependent REases.

### DNA/RNA metabolic enzymes associated with TGT

We found additional DNA metabolic enzymes such as DNA integrase, transposase, DNA helicase, PD-D/EXK nuclease (Cas4-like exonuclease), DNA MTase, resolvase, and toxin-antitoxin pair adjacent to the *tgt* gene. The most frequently found nucleases are PLD-family nuclease or Cas4-like nuclease in conjunction with a DNA helicase. We also found DNA/RNA metabolic enzymes next to a QueC-GTP cyclohydrolase-tRNA guanosine transglycosylase gene cluster, including a DNA primase, a Type I R-M system, a Zn-dependent hydrolase (amino deaminase) and a Piwi-domain protein that may encode a RNA-guided interference system^25^. An alternative explanation is that host-encoded restriction system, protease (peptidase) to degrade phage replication enzymes and morphogenesis proteins, or RNase to destroy preQ_0_-tRNA intermediate may be located elsewhere in the genome. The modification-dependent restriction system may have evolved independently as in the case of McrA positioned on a defective prophage^42^ and PvuRts1I located on a plasmid^20^. In future study, close inspection of the restriction locus (the “immigration control region”) harboring Type I R-M system and Type IV systems (McrBC, Mrr, or GmrSD) in sequenced *E*. *coli* genomes may provide a hint on newly acquired Type IV restriction system displacing McrBC, Mrr, and GmrSD^18, 19, 43^. Alternatively, the 15–16 kb “immigration control region” could be amplified by long-range PCR, sequenced and potential ORFs interrogated among the restriction-proficient WT strains.

### Phage 9 g *pac* site

Phage 9 g terminase makes a major cut at the *pac* site (immediately downstream of the two 21-bp direct repeats). Direct run-off sequencing of the viral genomic DNA indicated a mixture of cleaved and uncleaved *pac* sequences, which suggested a “headful” packaging mechanism (reviewed in refs 32 and 33). In phage “headful” DNA packaging, the first cut at the *pac* site (initiation of packaging) is relatively precise, but the subsequent cuts to terminate DNA packaging are non-specific. To reflect the true nature of the physical end of phage 9 g genome, we suggest to renumber the phage DNA coordinate in GenBank.

REases with A/T only recognition sequences such as DraI and PsiI can be used for diagnostic cleavage of the *pac*-site DNA fragment. Phage 9 g genomic DNA may contain more than 3 kb extra DNA possibly as terminal redundant repeat as the result of “headful” packaging mechanism. This terminal repeat might be edited out (deleted) by DNA sequencing assembly/editing software during shot-gun sequencing and assembly^13^. The extra terminal repeat DNA (~3–10% terminal redundancy depending on the phage genomes that had been studied to date) probably ensures that the viral DNA can form a circular molecule by homologous recombination and successfully overcome host-encoded exonucleases during phage DNA ejection into periplasm and transported into the cytoplasm^33^.

### TGT-like enzyme in phage and bacteria

When the aa sequence of phage 9 g TGT (predicted) was used in a BlastP search, many TGT homologs were found in bacteriophage genomes, in over 200 bacteria and archaea genomes, and in uncultured microorganisms (these homologs were annotated as hypothetical proteins, aTGT or queuosine tRNA ribosyltransferase with 20% to 98% aa sequence identity). It was speculated that the dG^+^ modified base was incorporated into phage DNA after DNA replication (i.e. the dG base in DNA is replaced by dG^+^ catalyzed by TGT enzyme post replication, similar to archaeosine modification in tRNA)^14^. Over-expression of dG^+^ modification enzymes (dG^+^ biosynthesis pathway) in *E*. *coli* host may enhance the level of dG^+^ modified bases in phage DNA. Cloning and expression of phage 9 g dG^+^ modification pathway along with DNA replication enzymes and characterization of these enzyme activities *in vitro* will help us understand more about phage restriction and modification, and the reconstitution of dG^+^ modification.

## Conclusion

*E*. *coli* phage 9 g contains the modified base dG^+^ in its genome (25–27% relative to dG) that renders the DNA resistant to approximately two third of Type II REases examined in this work. Restriction fragments with blunt ends or sticky ends derived from phage 9 g can be ligated by T3 and T4 DNA ligases. *E*. *coli* DNA ligase is able to ligate the sticky ends efficiently, but not blunt ends. Phage 9 g restriction fragments (SspI fragments) can be degraded by a number of DNA exonucleases. Phage 9 g DNA can be further modified by the frequent adenine methyltransferase EcoGII and a few other ^m5^C MTases, such as M.AluI, M.CviPI, and M.HhaI. This study provides a proof-of-concept experiment that multiple base modifications could in principle be introduced into phage 9 g. Phage 9 g DNA can serve as a template for PCR with Phusion® and Q5® DNA Polymerases. Some WT *E*. *coli* strains isolated from the environment can attenuate phage 9 g infection possibly by Type II restriction systems targeting A/T only recognition sequence, by modification-dependent restriction, or by other unknown phage attenuation mechanisms. Phage 9 g resistant NEB 10β mutants have been isolated that are resistant to both phage 9 g and λ infection. GAT-QueC homologs are found in five *Enterobacteria* and *Pseudomonas* phages suggesting that these phages also contain dG^+^ modification. dG^+^ modification and restriction enzymes have been found in bacterial genomic islands, suggesting phages may have acquired the dG^+^ synthesis pathway by phage transduction and horizontal gene transfer. Gene neighborhood analysis of DNA/RNA metabolic enzymes adjacent to TGT or QueC showed co-localization of PLD family nuclease, Cas4-like nuclease, DNA helicase, Type I R-M system, RNA-guided interference system (Piwi-domain protein), DNA repair enzyme, integrase and transposase associated with mobile genetic elements. Phage 9 g teminase binding and cleavage site (*pac* site) was mapped by direct sequencing of the phage DNA and restriction mapping.

## Methods

### *E. coli* host strains, plasmid DNA, restriction and modification enzymes

*E*. *coli* lab strains C2566 (B strain), and K strains NEB Turbo and 10β were from Dr. Lise Raleigh’s strain collection at New England Biolabs (NEB). WT *E*. *coli* strains (non-pathogenic) from human and animal sources were from Dr. Mehmet Berkmen’s private collection (NEB), courtesy of Professor David Gordon, Australian National University (ANU) College of Medicine, Biology & Environment. The restriction and modification enzymes and phage λ DNA utilized in this study are commercially available from NEB. DraI purchased from other suppliers (Thermo-Fisher, Promega) was also used in restriction of phage 9 g DNA. Restriction digestions, methylation, PCR, and exonuclease digestion were carried out according to the protocols recommended by the manufacturer (NEB). In most restriction digestions we used 10-fold over-digestion when REases were available in concentrated format at 10 to 20 units/μl (i.e. 40 units to digest 4 μg of phage 9 g DNA in 1 h at the recommended temperature). A few REases were only available in low concentration (2–5 units/μl) and restriction digestions were carried out at 2.5 to 6.3-fold over-digestion. DNA ligation reactions catalyzed by *E*. *coli*, T3, and T4 DNA ligases were carried out at 25 °C for 4 h. Ligation reactions of Taq and 9°N DNA ligase and restriction fragments were performed at 45 °C and 60 °C, respectively, for 4 h. To test sensitivity to exonucleases, two units of Bal-31 nuclease was used to digest 1 μg of phage 9 g and λ SspI restriction fragments at 30 °C for 30 min. Twenty units of λ exonuclease, T5 exonuclease, T7 exonuclease, *E*. *coli* exonuclease V were used to digest 1 μg of phage 9 g and phage λ SspI restriction fragments for 10, 20, and 30 min at 37 °C. T7 exonuclease digestion was carried out at 25 °C for 10–30 min.

### Phage 9 g DNA transfection, purification and agarose gel electrophoresis

Phage 9 g DNA was obtained from Dr. Richard J. Roberts (NEB). Phage 9 g viral DNA was transfected into two *E*. *coli* K strains NEB Turbo and 10β. Chemical competent cells (100 μl) were mixed with 50 ng of viral DNA and incubated at 4 °C for 30 min. After heat shock treatment (30 seconds at 42 °C), fresh cells (200 μl) were added, followed by addition of 4 ml of soft agar (melted and pre-warmed at 55 °C). The entire mixture was plated on Rich agar plates and incubated at 37 °C overnight to obtain single phage plaques. NEB 10β was used as the host for liquid infection and phage 9 g DNA was purified by standard protocols^44^. For LC-MS analysis of the dG^+^ content, the genomic DNA was gel-purified from 0.8% agarose gel followed by spin-column purification (Wizard mini-column, Promega). Restricted, ligated, exonuclease-digested, or PCR-amplified DNAs were analyzed by agarose gel (1%) electrophoresis.

### Nucleoside analysis

DNA samples (5 μg) were digested to nucleosides based on the method described by Hashimoto *et al*.^45^ using a proprietary blend of nuclease(s) and phosphatase(s) (NEB). Nucleoside analysis was performed on an Agilent LC/MS System 1200 Series instrument equipped with a G1315D diode array detector and a 6120 Single Quadrupole Mass Detector operating in positive (+ESI) and negative (−ESI) electrospray ionization modes. LC was carried out on a Waters Atlantis T3 column (4.6 × 150 mm, 3 μm) with a gradient mobile phase consisting of 10 mM aqueous ammonium acetate (pH 4.5) and methanol. MS data acquisition was recorded in total ion chromatogram (TIC) mode. Each nucleoside was identified as follows: dC [M + H]^+^ 228.1 and [M-H]^−^ 226.2; dG [M + H]^+^ 268.1 and [M-H]^−^ 266.1; dT [M + H]^+^ 243.1 and [M-H]^−^ 241.1; dA [M + H]^+^ 252.1 and [M-H]^−^ 250.1; d^5m^C [M + H]^+^ 242.1 and [M-H]^−^ 240.1; d^*N*6m^A [M + H]^+^ 266.1 and [M-H]^−^ 264.1; and dG^+^ [M + H]^+^ 309.1 and [M-H]^−^ 307.1. The relative abundance of each nucleoside was determined by dividing the UV absorbance by the corresponding extinction coefficient at 260 nm. dG^+^ was quantified on the basis of the PreQ_0_ extinction coefficient ε264 = 10,090 M^−1^ cm^−1^ The relative abundance of each nucleoside was normalized to dT (set as 1)^14^.

### Bioinformatic analysis of protein sequences

BlastP was used to search homologous proteins in GenBank via NCBI Blast server. PROMAL3D was used to perform multiple amino acid sequence alignments and secondary structure predictions^46^. NEBCutter software (http://tools.neb.com/REBsites/) was used to perform computer-generated restriction digestion patterns of phage 9 g DNA^47^. SEED Viewer was used to analyze adjacent genes in bacterial genomes^48^. A listing of confirmed and predicted Type I specificities can be found in REBASE^34^.

### Phage plating (phage spot test, phage restriction test)

*E*. *coli* host cells were grown in phage broth to mid-exponential phase. Cells were plated with soft agar on rich media plates. Five μl of diluted phage 9 g (10^3^ to 10^6^ pfu/ml) were spotted onto the cell lawn and the plates were incubated at 37 °C overnight. Alternatively, diluted phage 9 g, λ_vir_, T4, and T4gt were plated on WT *E*. *coli* hosts to test for plaque-forming units and restriction level.

### WT *E. coli* cell extract preparation and nuclease activity assay

Ten ml of overnight cell culture in LB was pelleted and resuspended in 1 ml of sonication buffer (50 mM NaCl, 20 mM Tris-HCl, pH 7.5, 1 mM DTT, and 1 mM EDTA). Cells were lysed by sonication and cell debris removed by centrifugation. Two to 5 μl of clarified cell lysate was incubated with mixed DNA substrates (1 μg of phage 9 g + 1 μg of λ BstEII restriction fragments) at 37 °C for 30 min. The digested DNA was further treated with Proteinase K and analyzed by agarose gel electrophoresis.

### DNA sequencing and run-off sequencing

Phage 9 g DNA (coordinate nt 25275 to 32833, ~7658 bp) was used as templates for direct sequencing using BigDye® cycle sequencing kit (ABI/Thermo-Fisher) by primer walking.

Primers in 19–24 nt length were synthesized by IDT. Terminase cut site (*pac* site) was determined by run-off sequencing of the *terB* upstream region of approximately 7.7 kb. SeqMan Pro of Lasergene (DNASTAR software package) was used for sequence analysis. AseI, DraI, and SspI digested phage 9 g DNA was purified by spin column purification and used as control DNA for run-off sequencing. Taq DNA polymerase introduces an extra A (adenine) using terminal nucleotide transferase activity when the DNA template is broken. In DNA sequencing using mixed templates with cut and uncut DNA molecules, A/C, A/T, or A/G doublets or extra high A peak are indication of broken templates (DNA ends created by REase, nicking endonuclease, phage terminase, or other DNA cleaving enzymes)^49^.

## Electronic supplementary material

Supplementary Information
