# Supplementary material for: Restriction and modification of deoxyarchaeosine (dG+)-containing phage 9 g DNA
Source: Sci Rep. 2017 Aug 21;7:8348. doi: 10.1038/s41598-017-08864-4 (PMC5567051; doi:10.1038/s41598-017-08864-4)
Supplement: Supplementary file 1 — Supplementary Information [file 41598_2017_8864_MOESM1_ESM.doc]

**Supplementary Material**

**Restriction and modification of deoxyarchaeosine (dG^+^)-containing phage 9g DNA**

Rebecca Tsai^1,2^, Ivan R. Corrêa Jr^1^., Michael Y. Xu^1,2^, and Shuang-yong Xu^1^*

1. New England Biolabs, Inc. 240 County Road, Ipswich, MA 01938, USA

2. Current address: Brandeis University, 415 South St., Waltham, MA 02453

*Corresponding author

Telephone: 978-380-7287

Fax: 978-921-1350

Email: [xus@neb.com](mailto:xus@neb.com)

**Supplementary Figure 1.** Computer generated restriction digestion of phage 9g DNA by NEBcutter (www.nebtools.neb.com/nebcutter). While NEBcutter generates all possible restriction fragments, the 1% agarose gels can positively identify fragments in the range of 0.3 to 10 kb. The predicted restriction patterns do not reflect the terminal end repeat sequence. The linear DNA sequence in GenBank imported into NEBcutter does not reflect the true nature of the physical ends (i.e. the *pac* fragment is also cleaved by the phage terminase).


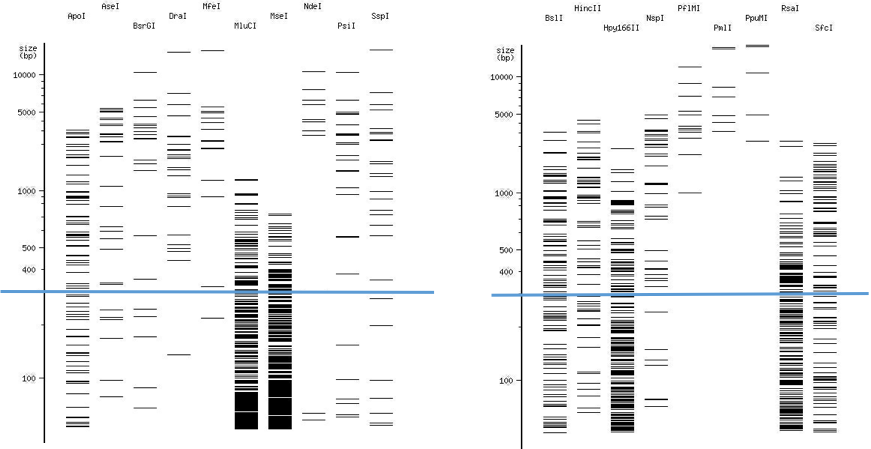


**Supplementary Figure 2**. Digestion of phage 9g and λ DNA by WT *E. coli* cell extracts.

Digestion of mixed DNA substrates (full-length phage 9g + λ BstEII restriction fragments) by WT *E. coli* cell extracts. The strain number is listed on top of each lane. + indicates both phage 9g and λ BstEII fragments were degraded (extensive smearing); ?, partial digestion by endonuclease/exonuclease (endo/exo) or inclusive result; -/+ (MB3634) indicates phage 9g DNA was mostly resistant while λ BstEII fragments were degraded to a significant extent; +/- (MB3635) indicates that full-length phage 9g DNA was degraded first while λ BstEII DNA fragments were degraded slower; --, uncut DNA substrates; M, 2-log DNA ladder (NEB).

**
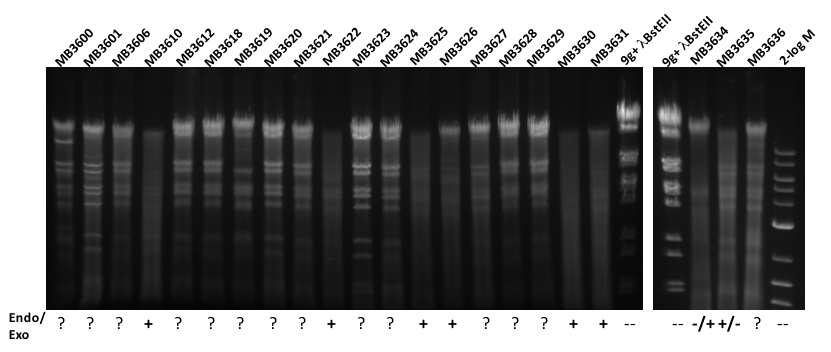
**

**Supplementary Figure 3.** DNA/RNA metabolic enzymes associated with QueC in some bacterial genomes. Left panel, the bacterial strain names and genomic region. Right panel, genomic loci with *queC* gene (red, boxed) as a “SEED” to examine the adjacent genes (ORFs). The DNA metabolic genes (predicted enzymes) and protease were shown on top of each gene. Two *G*. *uraniireducens* genomes contain a preQ_0_ biosynthesis pathway (three-gene cluster) next to a Type I R-M system and a putative RNA-guided RNA interference system (i.e. Piwi-domain protein, Pin nuclease).


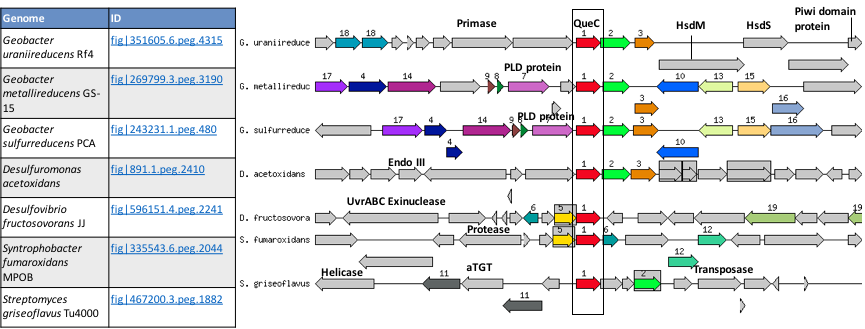


**Supplementary Figure 4.** Phage 9g resistant *E. coli* mutants and phage spot test on the mutant cells.

1. NEB 10β colonies outgrown from a lysed cell lawn. Each dark dot was formed by a phage-resistant mutant colony.
2. NEB 10β cell lawn spotted with phage 9g, λ, and T4 at three dilutions (10^-2^, 10^-4^, 10^-6^).
3. D. Phage spot test on NEB 10β mutant cell lawns (two independent mutants from spontaneous mutation). Five μl of the diluted phage stock were spotted on the cell lawns.

**Supplementary Figure 5.** PROMALS3D multiple sequence alignment of phage-encoded GAT-QueC (7-cyano-7-deazaguanine synthases, 52% to 98% aa sequence identity) which catalyzes the conversion of 7-deaza-7-carboxyguanine to preQ_0_. The number (5 to 9) indicates the level of conservation. Secondary structure prediction: h, α-helix; e, β-sheet. Sequence in box, highly conserved aa residues among the six aa sequences.

Conservation: 99 9 9 5 9 99999 9 99 9 5

YP_009285849_phage_NP1_ 1 MCAIIGALVWGLTTPEARSQANGLLNHIVAHSHERGRDGRGFLTNYGD---DIVIEKSTDRKDAKDW-TP 66

ALH23790_phage_PaMx25_ 1 MCAIIGALVWGLTTPDTRSQANGLLNHIIQQSHERGRDGRGFLTNYGD---DIVIEKSTERKEGKDW-HP 66

YP_009032329_phage_9g_ 1 MCSIFGVIT---NGQPESLIIRNDIEDLIKASIKRGRDGLGVRFTHGDHSMHPQRIVSVGKETSLERLVK 67

YP_009219314_phage_JenK1_ 1 MCSIFGVIT---NGQPESLIIRNDIEDLIKASISRGRDGLGVRFTHGDHSMHPQRIVSVGKKTSLERLVK 67

YP_009220006_phage_JenP1_ 1 MCSIFGVIT---NGQPESLIIRNDINDLIKASINRGRDGLGVRFTHGDHSMHPQRIVSVGKETSLERLVK 67

YP_009216973_phage_JenP2_ 1 MCSIFGVIT---NGQPESLIIRNDIDDLIKASINRGRDGLGVRFTHGDHSIHPQRIVSVGKETSLERLVK 67

Consensus_aa: MCtIhGhlh...ss...p.bhps.lpcll..ShpRGRDG.Gh.hs@GD...c...b.Sh.+cptb-..h.

Consensus_ss: eeeeee hhhhhhhhhhhhhhhhh eeeeee eeeeeee hhhhhhhhh

Conservation: 9 5 9 9 9 9 9999999 5 59 99999 9 99999999999 599 9 999

YP_009285849_phage_NP1_ 67 VEFFKS---DVSSATFISNLRAEPTTEYVADKSQDDQQPYSAGHWSIVHNGTIANDKALRTGKVPTRIDS 133

ALH23790_phage_PaMx25_ 67 VEFFKG---DVESATFISNLRAEPTTEYVANKSQDDQQPYSAGHWSIVHNGTIANDKALRTGKVPTRIDS 133

YP_009032329_phage_9g_ 68 VGRIASHAGAIGSFTMIGNARAEPTTEWIVDKNEWDQQPYHMDDWTIVHNGTIANDKDIRTYALQTKIDS 137

YP_009219314_phage_JenK1_ 68 VGRIVSHAGAIGSFTMIGNARAEPTTEWVVDKNEWDQQPYHMDGWTIVHNGTIANDKDLRTYALQTKIDS 137

YP_009220006_phage_JenP1_ 68 VGRIASHAGAIGSFTMIGNARAEPTTEWVVDKNEWDQQPYHMDGWTIVHNGTIANDKDLRTYALQTKIDS 137

YP_009216973_phage_JenP2_ 68 VGRIASHAGAIGSFTMIGNARAEPTTEWVVDKNEWDQQPYHLDSWTIVHNGTIANDKDLRTYALQTKIDS 137

Consensus_aa: V..h.t...sl.ShThItNhRAEPTTE@VhsKsp.DQQPYphs.WoIVHNGTIANDKsLRT..l.T+IDS

Consensus_ss: hhhh eeeeeeeeeeeee ee eeeee eee hhhhhhhhh h

Conservation: 999 9 9 9 9 9 9 999995999999 9 999999 9 9

YP_009285849_phage_NP1_ 134 AAIAEVLDANRCDG-----SNAYRTALHFIEGVKKLKGSYAILATHSEQKNHLLVAANYRPIWFVVTEVG 198

ALH23790_phage_PaMx25_ 134 AAIAEVLDANRCDG-----SNAYRTALHFIEGVKKLKGSYAILATHSEQKNHLLVAANYRPIWFVVTEVG 198

YP_009032329_phage_9g_ 138 AAIVEQLAECTLSADEMCLSDFNYLYSVFHEVVRKLKGSFAILATHDSFPGCVFTACNYRPIWIGKTDTG 207

YP_009219314_phage_JenK1_ 138 AAIVEQLAECTLSADEMCLSDFNYLYSVFHEVVRKLKGSFAILATHDSFPGCVFTACNYRPIWIGKTDTG 207

YP_009220006_phage_JenP1_ 138 AAIVEQLAECTLSADEMCLSDFNYLYSVFHEVVRKLKGSFAILATHDSLPGYVFAACNYRPIWIGKTQTG 207

YP_009216973_phage_JenP2_ 138 AAIVEQLAECTLSTDEMCLSDLNYLYNVFHEVVRKLKGSYAILATHDSIPGYVFAACNYRPIWIGKTHTG 207

Consensus_aa: AAIhE.Ls.sphst.....Ssh..hh.hFhEsV+KLKGS@AILATHspb.shlhhAtNYRPIWhs.TchG

Consensus_ss: hhhhhhhhhhhhhhhhhh hhhhhhhhhhhhh eeeeeee eeeeee eeeeee e

Conservation: 999 9 9 9999 999 99 999 9

YP_009285849_phage_NP1_ 199 VFFASARHYFPDNMTPRMVDPYSVMSFYFSDHLEIRGESLYDVGARSKALVVCSGGLDSVVAATYVKKVI 268

ALH23790_phage_PaMx25_ 199 VFFASARHYFPDNMTPRMVDPYSVMSFYFSDHLEIRGESLYDVGARSKALVVCSGGLDSVVAATYVKKVI 268

YP_009032329_phage_9g_ 208 MYFASQEDMLPN--RAIKQMLKPYSCNVVTSTDVCNEVDLIPNKQTQRALVVASGGMDSTVAAQMCKSNG 275

YP_009219314_phage_JenK1_ 208 MYFASQEDMLPT--RAIKQMLKPYSCNVVTSTDVCNEVDLIPNKQTQRALVVASGGMDSTVAAQMCKSNG 275

YP_009220006_phage_JenP1_ 208 MYFASQEDMLPT--RAIKQMLKPYTCNVVTSTDVCNEVDLIPNKQTQRALVVASGGMDSTVAAQMCKSNG 275

YP_009216973_phage_JenP2_ 208 MYFASQEDMLPT--RAIKQMLKPYTCNVVTSTGVCSEVDLIPNKQTQRALVVASGGMDSTVAAQMCKSNG 275

Consensus_aa: h@FAS.cchhPs..ps.b...bsh.t.hhosh..hp..sLhss..pp+ALVVtSGGhDShVAAphhKps.

Consensus_ss: eeee eeee eee hhh hhhhhhhhhhhh

Conservation: 5 99 9 99 999 9 9 9 599 99 9 9 9999 9 999999999999999

YP_009285849_phage_NP1_ 269 GVDTELIHFCYGSRAEGPEVIAVQAAADALGVPCTVFPLPVYKPSDSPLLQADSKIAGGEEGAEFAHEWV 338

ALH23790_phage_PaMx25_ 269 GVETELIHFCYGSRAEGPEVIAVQAAADAIGVPCTVFPLPVYKPSDSPLLQADSKIAGGEEGAEFAHEWV 338

YP_009032329_phage_9g_ 276 -LDVTLINFQYGCRAETNELYAIRKIAEVMGVPLIEFPIPIYDKKDSPLFDQDAAIAGGEEGAEFAHEWV 344

YP_009219314_phage_JenK1_ 276 -LDVTLINFQYGCRAETNELYAIRKIAEVMEVPLVEFPIPIYDKKDSPLFDQDAAIAGGEEGAEFAHEWV 344

YP_009220006_phage_JenP1_ 276 -LDVTLINFQYGCRAETNELYAIRKIAEVMEVPLIEFPIPIYDKKDSPLFDQDAAIAGGEEGAEFAHEWV 344

YP_009216973_phage_JenP2_ 276 -LDVTLINFQYGCRAETNELKAIRKIAEVMEVPLVEFPIPIYDKKDSPLFDQDAAIAGGEEGAEFAHEWV 344

Consensus_aa: .l-hpLIpF.YGtRAEssElhAlp.hA-hh.VPhh.FPlPlYc.pDSPLhp.Dt.IAGGEEGAEFAHEWV

Consensus_ss: eeeeeee hhhhhhhhhhhhh eeeeeeehhh hhhhhhhhh hh ee

Conservation: 99999 99999999999999 999999999999999999999 99 9999999999 9 9999999

YP_009285849_phage_NP1_ 339 PARNLLLLSVATAYAEANGFDTIVLGNNLEEAGAYPDNEPEFIAKFNDLLPFAVGDGKRMRVMMPVGNLM 408

ALH23790_phage_PaMx25_ 339 PARNLLLLSVATAYAEANGFDTIVLGNNLEEAGAYPDNEPEFIAKFNDLLPFAVGDGKRMRVMMPVGNLM 408

YP_009032329_phage_9g_ 345 PARNLVMLSVATAYAEANGFDYIVLGNNLEEAGAYPDNEPEFINRFNQVLPFAVGDGKRVEVLMPVGNLM 414

YP_009219314_phage_JenK1_ 345 PARNLVMLSVATAYAEANGFDYIVLGNNLEEAGAYPDNEPEFINRFNQVLPFAVGDGKRVEVLMPVGNLM 414

YP_009220006_phage_JenP1_ 345 PARNLVMLSVATAYAEANGFDYIVLGNNLEEAGAYPDNEPEFINRFNQVLPFAVGDGKRVEVLMPVGNLM 414

YP_009216973_phage_JenP2_ 345 PARNLVMLSVATAYAEANGFDYIVLGNNLEEAGAYPDNEPEFINRFNQVLPFAVGDGKRVEVLMPVGNLM 414

Consensus_aa: PARNLlhLSVATAYAEANGFDhIVLGNNLEEAGAYPDNEPEFIs+FNplLPFAVGDGKRhcVhMPVGNLM

Consensus_ss: ehhhhhhhhhhhhhhhh eeeeeee hhhhhhhhhhhhh eeeee

Conservation: 99999 9 9 9999 999999 9 99999999 995999 99 999 9 59

YP_009285849_phage_NP1_ 409 KHEIVELGHRIGAPLDKTWSCYRAGEQHCGTCGPCYMRRTAFEINGLEEVITYANEKE 466

ALH23790_phage_PaMx25_ 409 KHEIVELGHRIGAPLDKTWSCYRAGEQHCGTCGPCYMRRTAFEINGLEEVITYANEKE 466

YP_009032329_phage_9g_ 415 KHEIVATGLRLGAPLEHTWSCYRNGNLHCGTCGPCMMRRTAFNINNAQEVIKYENEE- 471

YP_009219314_phage_JenK1_ 415 KHEIVATGLRLGAPLEHTWSCYRNGDLHCGTCGPCMMRRTAFNINNAQEVIKYESEE- 471

YP_009220006_phage_JenP1_ 415 KHEIVATGLRLGAPLEHTWSCYRNGDLHCGTCGPCMMRQTAFNINNAQEVIKYENEE- 471

YP_009216973_phage_JenP2_ 415 KHEIVATGLRLGAPLEHTWSCYRNGDLHCGTCGPCMMRRTAFNINNAQEVIKYENEE- 471

Consensus_aa: KHEIV.hGhRlGAPL-+TWSCYRsG-bHCGTCGPChMRRTAFpINshpEVIpY.NEc.

Consensus_ss: hhhhhhhhhh hhhee hhhhhhhhhhhh

**Supplementary Figure 6.** DNA Run-off sequencing using partially cut or completely cut phage 9g DNA as templates.

1. Run-off sequencing using cut and uncut mixed templates (~33% cut + ~67% uncut in this region). The up arrow indicates the bottom-strand nick (cleavage) where the higher A peak and overall peaks drop-off are detected.
2. Run-off sequencing using cut and uncut mixed templates (~67% cut + ~33% uncut in this region)
3. Run-off sequencing using completely digested templates (>99%% cut) and total sequence peaks drop-off.

**Supplementary Figure 7.** Schematic diagrams of the *pac* site cleavage and initiation of “headful” DNA packaging.

1. Terminase cleavage site near the *pac* sequence mapped by DNA run-off sequencing. The major cut (bottom-strand) was mapped to nt 28243. Top-strand cut site is not determined.
2. Proposed DNA packaging direction in “headful” packaging and serial encapsidation process. Two circles, terminase large and small subunits (the exact subunit stoichiometry is unknown). In this model, the late morphogenesis genes are packaged first and the early genes are packaged last. The first terminase cleavage near the *pac* site is relatively precise. The second or third terminase cut sites are probably non-specific. The procapsid where terminase binds and initiates DNA packaging is not shown. The large empty arrow indicates the direction of DNA packaging. The red box represents the terminal redundant repeat of approximately 3.2 kb.

**Supplementary Figure 8.** DraI restriction digestion of phage 9g DNA.

1. Phage 9g DNA was digested by DraI (20, 10, and 5 units, respectively) from three suppliers and cleavage products were analyzed on a 0.8% agarose gel. Arrows 1, 2, and 3 indicate a shifted slow migrating band, *pac* restriction fragments (a doublet of ~8.4 kb), and a DraI fragment encompassing nt 1 and nt 56702 (~2.2 kb), respectively.
2. Predicted DraI restriction fragments from GenBank sequence NC_024146. The fragments marked by X were not detected in the actual gel. See the main text for explanations. The small fragments (#25 and #26) were too small to be detected on the 0.8% agarose gel.
3. Schematic diagram of the DraI cut sites in the linear phage 9g DNA. Note: the left end nt 1 and right end nt 56702 are physically continuous. The actual broken point of the linear phage DNA is at or near the *pac* site (cut by the phage terminase during phage DNA packaging).

Note: the missing 5.2 kb right end of the viral DNA is found to be 8.4 kb. Thus the terminal end redundant repeat (circular permutation) is calculated as 8.4 – 5.2 = ~3.2 kb.

**Supplementary Figure 9.** Restriction mapping of phage 9g *pac* fragments.

Left panel, NEBcutter-generated restriction patterns for AclI, AflIII, ApoI, AseI, DraI, MfeI, MluCI, PsiI, RsaI, and SspI-HF on a hypothetical agarose gel (0.7%). Terminase cleavage at the *pac* site is not accounted for in these restriction patterns.

Right panel, restriction digestion of phage 9g DNA by ten individual REases and the cleavage products were analyzed on a long 0.8% agarose gel (i.e. the DNA was cleaved by terminase and REase). AseI *pac* fragment: nt 26974-28243 = 1269 bp (*). DraI *pac* fragment: nt 19879-28243 = 8364 bp (*). MluCI *pac* fragment: nt 27176-28243 = 1067 bp (*). PsiI *pac* fragments: nt 25215-28243 = 3028 bp (*). RsaI-*pac* fragment: nt 26291-28243 = 1952 bp (*, this fragment overlaps with another fragment (nt 51486-53478) to form a doublet). SspI *pac* fragments: nt 27610-28243 = 633 bp (*, this small fragment overlaps with another fragment (nt 26978-27610) to form a doublet). The *pac* fragments in other restriction digestions were difficult to identify due to partial digestions. The dashed line serves as a reference point for the predicted and actual size DNAs.

**Supplementary Table 1.** Phage 9g DNA susceptibility to Type II restrictions.

We classified restriction digestions into four major groups: C, for complete digestion; P, for partial digestion; VP, for very partial digestion (i.e. the full-length DNA substrate remains intact, only a few faint bands were visible); x, for completely resistant to digestion. All restriction digestions were carried out in the recommended buffer and optimal temperature. A total of 214 REases were tested in restriction of phage 9g DNA. 31 REases (~14.5%) completely digested the DNA; Another 31 REases (~14.5%) generated partial digestion. 27 REases (~12.6%) displayed very partial digestion. The remaining 58.4% of REases failed to cleave dG^+^-modified 9g DNA. Phage 9g genome has no restriction sites for 18 REases (13 six-base cutters, 1 seven-base cutter, 4 eight-base cutters). A few modification-dependent Type IIM and IV REases did not cleave phage 9g DNA due to the lack of ^5m^C or ^N4m^C modification.

| **REase** | **Recognition sequence** | **# of G'S** | **Digestion** | **Cleavage Product*** |
| --- | --- | --- | --- | --- |
| AatII | GACGTC | 4 | x | - |
| AccI | GTMKAC | 2 to 4 | p | >12 |
| Acc65I | GGTACC | 4 | (no site) |  |
| AciI | CCGC | 4 | c | 14 |
| AclI | AACGTT | 2 | c | >15 |
| AcuI | CTGAAGN | 3 | x | - |
| AfeI | AGCGCT | 4 | (no site) |  |
| AflII | CTTAAG | 2 | vp | 3 |
| AflIII | ACRYGT | 2 to 4 | c | >12 |
| AgeI-HF | ACCGGT | 4 | x | - |
| AhdI | GAC N5 GTC | 4 | x | - |
| AleI | CAC N4 GTG | 4 | vp | 2 |
| AluI | AGCT | 2 | c | 11 |
| AlwI | GGATC | 3 | x | - |
| AlwNI | CAG N3 CTG | 4 | p | 7 |
| ApaI | GGGCCC | 6 | x | - |
| ApaLI | GTGCAC | 4 | (no site) |  |
| ApeKI | GCWGC | 4 | c | >12 |
| ApoI | RAATTY | 0 to 2 | p | >26 |
| AscI | GGCGCGCC | 8 | (no site) |  |
| AseI | ATTAAT | 0 | c | >14 |
| AsiSI | GCGATCGC | 6 | x |  |
| AvaI | CYCGRG | 4 to 6 | x | - |
| AvaII | GGWCC | 4 | x | - |
| AvrII | CCTAGG | 4 | (no site) |  |
| BaeI | AC N4 GTAYC | 3 to 4 | p | 4 |
| BaeGI | GKGCMC | 4 to 6 | x | - |
| BamHI-HF | GGATCC | 4 | x | - |
| BanI | GGYRCC | 4 to 6 | x | - |
| BanII | GRGCYC | 4 to 6 | x | - |
| BbsI | GAAGACN2 | 3 | x | - |
| BbvI | GCAGCN8 | 4 | c | >13 |
| BbvCI | CCTCAGC | 5 | x | - |
| BccI | CCATCN4 | 3 | x | - |
| BceAI | ACGGCN12 | 4 | vp | 4 |
| BcgI | CGA N6 TGCN12 | 4 | x | - |
| BciVI | GTATCCN6 | 3 | x | - |
| BclI | TGATCA | 2 | x | - |
| BcoDI | GTCTCN1 | 3 | x | - |
| BfaI | CTAG | 2 | p | >14 |
| BfuAI | ACCTGCN4 | 4 | c | >15 |
| BfuCI | GATC | 2 | x | - |
| BglI | GCC N5 GGC | 6 | x | - |
| BisI (Type IIM) | G(5m)CNGC | 4 | x |  |
| BglII | AGATCT | 2 | x | - |
| BlpI | GCTNAGC | 4 | x | - |
| BmgBI | CACGTC | 4 | x | - |
| BmrI | ACTGGGN5 | 4 | vp | 4 |
| BmtI-HF | GCTAGC | 4 | (no site) |  |
| BpmI | CTGGAGN16 | 4 | x | - |
| Bpu10I | CCTNAGC | 4 | vp | 9 |
| BpuEI | CTTGAGN16 | 3 | x | - |
| BsaI-HF | GGTCTCN1 | 4 | x | - |
| BsaAI | YACGTR | 2 to 4 | c | >13 |
| BsaBI | GAT N4 ATC | 2 | x | - |
| BsaHI | GRCGYC | 4 to 6 | x | - |
| BsaJI | CCNNGG | 4 | p | 5 |
| BsaWI | WCCGGW | 4 | x | - |
| BsaXI | ACN5CTCCN10 | 4 | x | - |
| BseRI | GAGGAGN10 | 4 | x | - |
| BseYI | CCCAGC | 5 | vp | 5 |
| BsgI | GTGCAGN16 | 4 | vp | 7 |
| BsiEI | CGRYCG | 4 to 6 | x | - |
| BsiHKAI | GWGCWC | 4 | vp | >13 |
| BsiWI | CGTACG | 4 | x | - |
| BslI | CC N7 GG | 4 | p | >17 |
| BsmI | GAATGCN | 3 | x | - |
| BsmAI | GTCTCN1 | 3 | x | - |
| BsmBI | CGTCTCN1 | 4 | x | - |
| BsmFI | GGGACN10 | 4 | x | - |
| BsoBI | CYCGRG | 4 to 6 | x | - |
| Bsp1286I | GDGCHC | 4 to 6 | vp | 4 |
| BspCNI | CTCAGN10 | 3 | x | - |
| BspDI | ATCGAT | 2 | x | - |
| BspEI | TCCGGA | 4 | x | - |
| BspHI | TCATGA | 2 | x | - |
| BspMI | ACCTGCN4 | 4 | p | >17 |
| BspQI | GCTCTTCN1 | 4 | x | - |
| BsrI | ACTGGN | 3 | p | >13 |
| BsrBI | CCGCTC | 5 | x | - |
| BsrDI | GCAATGNN | 3 | p | >15 |
| BsrFI | RCCGGY | 4 to 6 | x | - |
| BsrGI-HF | TGTACA | 2 | p | >15 |
| BssHII | GCGCGC | 6 | (no site) |  |
| BssS_a_I | CACGAG | 4 | x | - |
| BstAPI | GCA N5 TGC | 4 | p | >13 |
| BstBI | TTCGAA | 2 | x | - |
| BstEII-HF | GGTNACC | 4 | x | - |
| BstNI | CCWGG | 4 | vp | 4 |
| BstUI | CGCG | 4 | c | >19 |
| BstXI | CCA N6 TGG | 4 | vp | 5 |
| BstYI | RGATCY | 2 to 4 | x | - |
| BstZI7I | GTATAC | 2 | c | 9 |
| Bsu36I | CCTNAGG | 4 | x | 13 |
| BtgI | CCRYGG | 4 to 6 | vp | 2 |
| BtgZI | GCGATGN10 | 4 | x | - |
| Bts_a_I | GCAGTGNN | 4 | x | - |
| BtsIMutI | CAGTGNN | 3 | p | 13 |
| BtsCI | GGATGNN | 3 | x | - |
| Cac8I | GCNNGC | 4 | c | 12 |
| ClaI | ATCGAT | 2 | x | - |
| CspcI | CAAN5GTGGN12 | 4 | vp | 5 |
| CviAII | CATG | 2 | p | >15 |
| CviKI-I | RGCY | 2 to 4 | p | >13 |
| CviQI | GTAC | 2 | c | >15 |
| DdeI | CTNAG | 2 | p | 9 |
| DpnI (Type IIM) | G(N6m)ATC | 2 | x | - |
| DpnII | GATC | 2 | x | - |
| DraI | TTTAAA | 0 | c | >16 |
| DraIII-HF | CAC N3 GTG | 4 | p | 11 |
| DrdI | GAC N6 GTC | 4 | x | - |
| EaeI | YGGCCR | 4 to 6 | x | - |
| EagI | CGGCCG | 6 | x | - |
| EarI | CTCTTCN1 | 3 | x | - |
| EciI | GGCGGAN11 | 5 | x | - |
| Eco53kI | GAGCTC | 4 | x | - |
| EcoNI | CCT N5 AGG | 4 | x | - |
| EcoO109I | RGGNCCY | 4 to 6 | x | - |
| EcoP15I (Type III) | CAGCAGN25 | 4 | vp | 3 |
| EcoRI-HF | GAATTC | 2 | x | - |
| EcoRV-HF | GATATC | 2 | x | - |
| FatI | CATG | 2 | c | >15 |
| FauI | CCCGCN4 | 5 | p | >12 |
| Fnu4HI | GCNGC | 4 | c | >12 |
| FokI | GGATGN9 | 3 | vp | 5 |
| FseI | GGCCGGCC | 8 | (no site) |  |
| FspI | TGCGCA | 4 | c | 10 |
| HaeII | RGCGCY | 4 to 6 | x | - |
| HaeIII | GGCC | 4 | x | - |
| HgaI | GACGCN5 | 4 | x | - |
| HhaI | GCGC | 4 | c | >13 |
| HincII | GTYRAC | 2 to 4 | p | >14 |
| HindIII-HF | AAGCTT | 2 | (no site) |  |
| HinfI | GANTC | 2 | x | - |
| HinP1I | GCGC | 4 | c | 7 |
| HpaI | GTTAAC | 2 | x | - |
| HpaII | CCGG | 4 | vp | 9 |
| HphI | GGTGAN8 | 3 | x | - |
| Hpy99I | CGWCG | 4 | p | 10 |
| Hpy166II | GTNNAC | 2 | c | >15 |
| Hpy188I | TCNGA | 2 | x | - |
| Hpy188III | TCNNGA | 2 | x | - |
| HpyAV | CCTTCN6 | 3 | x | - |
| HpyCH4III | ACNGT | 2 | p | >15 |
| HpyCH4IV | ACGT | 2 | c | 10 |
| HpyCH4V | TGCA | 2 | c | 8 |
| KasI | GGCGCC | 6 | x | - |
| KpnI-HF | GGTACC | 4 | (no site) |  |
| MboI | GATC | 2 | x | - |
| McrBC (Type IV) | Pu(5m)C-N40-3000-Pu(5m)C | 2 to 4 | x |  |
| MboII | GAAGAN8 | 2 | x | - |
| MfeI | CAATTG | 2 | p | 7 |
| MluI-HF | ACGCGT | 4 | p | 9 |
| MluCI | AATT | 0 | c | 8 |
| MlyI | GAGTCN5 | 3 | x | - |
| MmeI | TCCRACN20 | 3 to 4 | x | - |
| MnlI | CCTCN7 | 3 | vp | 5 |
| MscI | TGGCCA | 4 | x | - |
| MseI | TTAA | 0 | c | 5 |
| MslI | CAY N4 RTG | 2 to 4 | vp | 6 |
| MspI | CCGG | 4 | p | 10 |
| MspJI (Type IIM) | (5m)CNNR | 1 to 2 | x | - |
| MwoI | GC N7 GC | 4 | p | 8 |
| NaeI | GCCGGC | 6 | x | - |
| NarI | GGCGCC | 6 | x | - |
| NciI | CCSGG | 4 to 5 | x | - |
| NcoI-HF | CCATGG | 4 | x | - |
| NdeI | CATATG | 2 | p | 6 |
| NgoMIV | GCCGGC | 6 | x | - |
| NheI-HF | GCTAGC | 4 | (no site) |  |
| NlaIII | CATG | 2 | c | >15 |
| NlaIV | GGNNCC | 4 | vp | 5 |
| NmeAIII | GCCGAGN20 | 5 | x | - |
| NotI-HF | GCGGCCGC | 8 | x | - |
| NruI-HF | TCGCGA | 4 | x | - |
| NsiI-HF | ATGCAT | 2 | x | - |
| NspI | RCATGY | 2 to 4 | p | >13 |
| PacI | TTAATTAA | 0 | c |  |
| PaeR7I | CTCGAG | 4 | x | - |
| PciI | ACATGT | 2 | p | 6 |
| PflFI | GAC N3 GTC | 4 | x | - |
| PflMI | CCA N5 TGG | 4 | x | - |
| PleI | GAGTCN4 | 3 | x | - |
| PluTI | GGCGCC | 6 | x | - |
| PmeI | GTTTAAAC | 2 | ?? |  |
| PmlI | CACGTG | 4 | vp | 2 |
| PpuMI | RGGWCCY | 4 to 6 | vp | 3 |
| PshAI | GAC N3 GTC | 4 | x | - |
| PsiI | TTATAA | 0 | c | >14 |
| PspGI | CCWGG | 4 | p | >15 |
| PspOMI | GGGCCC | 6 | x | - |
| PspXI | VCTCGAGB | 4 to 6 | x | - |
| PstI-HF | CTGCAG | 4 | x | - |
| PvuI-HF | CGATCG | 4 | x | - |
| PvuII-HF | CAGCTG | 4 | x | - |
| RsaI | GTAC | 2 | c | 11 |
| RsrII | CGGWCCG | 6 | (no site) |  |
| SacI-HF | GAGCTC | 4 | x | - |
| SacII | CCGCGG | 6 | x | - |
| SalI-HF | GTCGAC | 4 | x | - |
| SapI | GCTCTTCN1 | 4 | x | - |
| Sau3AI | GATC | 2 | x | - |
| Sau96I | GGNCC | 4 | x | - |
| SbfI-HF | CCTGCAGG | 6 | (no site) |  |
| ScaI-HF | AGTACT | 2 | (no site) |  |
| ScrFI | CCNGG | 4 | vp | >14 |
| SexAI | ACCWGGT | 4 | vp | 2 |
| SfaNI | GCATCN5 | 3 | vp | 3 |
| SfcI | CTRYAG | 2 to 4 | p | 10 |
| SfiI | GGCC N5 GGCC | 8 | (no site) |  |
| SfoI | GGCGCC | 6 | x | - |
| SgrAI | CRCCGGYG | 6 to 8 | x | - |
| SmaI | CCCGGG | 6 | x | - |
| SmlI | CTYRAG | 2 to 4 | vp | 4 |
| SnaBI | TACGTA | 2 | (no site) | 5 |
| SpeI-HF | ACTAGT | 2 | (no site) |  |
| SphI-HF | GCATGC | 4 | (no site) |  |
| SspI-HF | AATATT | 0 | c | >16 |
| StuI | AGGCCT | 4 | x | - |
| StyI-HF | CCWWGG | 4 | x | - |
| StyD4I | CCNGG | 4 | vp | 9 |
| SwaI | ATTTAAAT | 0 | c |  |
| TaqI (alpha) | TCGA | 2 | c | >12 |
| TfiI | GAWTC | 2 | x | - |
| TseI | GCWGC | 4 | p | >13 |
| Tsp45I | GTSAC | 3 | x | - |
| TspMI | CCCGGG | 6 | x | - |
| TspRI | NNCASTGNN | 3 | p | 8 |
| Tth111I | GACNNNGTC | 4 | x | - |
| XbaI | TCTAGA | 2 | x | - |
| XcmI | CCA N9 TGG | 4 | vp | 12 |
| XhoI | CTCGAG | 4 | x | - |
| XmaI | CCCGGG | 6 | x | - |
| XmnI | GAA N4 TTC | 2 | x | - |
| ZraI | GACGTC | 4 | x | - |
|  |  |  |  |  |
| **Statistics** |  |  |  | * 0.3 to 12 kb products |
| # of Type IIM: 3 | |  |  | were counted from |
| REs without recognition site: 18 |  |  |  | 1% agarose gels. |
| # of Type III RE: 1 | |  |  |  |
| # of Type II RE: 213 | |  |  |  |
| 31, completely cut (14.5%) | | |  |  |
| 31, partially cut (14.5%) | |  |  |  |
| 27, very partial (12.6%) | |  |  |  |

**Supplementary Table 2.** Base call (nt) differences in phage 9g DNA sequence (from nt 25275 to nt 32833)

| nt sequence position* | Direct sequencing using phage 9g DNA as template | GenBank accession # NC_024146 |
| --- | --- | --- |
| 25837 | T | C |
| 25854 | G | A |
| 26474 | A | G |
| 26480 | A | G |
| 28519 | T | C |
| 28566 | G | C |
| 28700 | A | G |
| 28753 | T | C |
| 30422 | +A** | missing 1 base call (deletion) |
| 30813 | G | A |

*nt position (coordinate) follows Genbank accession number NC_024146.

**a stretch of 5As.
